# *In vivo* and *in silico* dynamics of the development of Metabolic Syndrome

**DOI:** 10.1371/journal.pcbi.1006145

**Published:** 2018-06-07

**Authors:** Yvonne J. W. Rozendaal, Yanan Wang, Yared Paalvast, Lauren L. Tambyrajah, Zhuang Li, Ko Willems van Dijk, Patrick C. N. Rensen, Jan A. Kuivenhoven, Albert K. Groen, Peter A. J. Hilbers, Natal A. W. van Riel

**Affiliations:** 1 Department of Biomedical Engineering, Eindhoven University of Technology, Eindhoven, The Netherlands; 2 Department of Pediatrics, Section Molecular Genetics, University Medical Center Groningen, University of Groningen, Groningen, The Netherlands; 3 Department of Medicine, Division of Endocrinology, Leiden University Medical Center, Leiden, The Netherlands; 4 Einthoven Laboratory for Experimental Vascular Medicine, Leiden University Medical Center, Leiden, The Netherlands; 5 Department of Human Genetics, Leiden University Medical Center, Leiden, The Netherlands; 6 Amsterdam Diabetes Center, Department of Vascular Medicine, Academic Medical Center, University of Amsterdam, Amsterdam, The Netherlands; Stanford University, UNITED STATES

## Abstract

The Metabolic Syndrome (MetS) is a complex, multifactorial disorder that develops slowly over time presenting itself with large differences among MetS patients. We applied a systems biology approach to describe and predict the onset and progressive development of MetS, in a study that combined *in vivo* and *in silico* models. A new data-driven, physiological model (MINGLeD: Model INtegrating Glucose and Lipid Dynamics) was developed, describing glucose, lipid and cholesterol metabolism. Since classic kinetic models cannot describe slowly progressing disorders, a simulation method (ADAPT) was used to describe longitudinal dynamics and to predict metabolic concentrations and fluxes. This approach yielded a novel model that can describe long-term MetS development and progression. This model was integrated with longitudinal *in vivo* data that was obtained from male APOE*3-Leiden.CETP mice fed a high-fat, high-cholesterol diet for three months and that developed MetS as reflected by classical symptoms including obesity and glucose intolerance. Two distinct subgroups were identified: those who developed dyslipidemia, and those who did not. The combination of MINGLeD with ADAPT could correctly predict both phenotypes, without making any prior assumptions about changes in kinetic rates or metabolic regulation. Modeling and flux trajectory analysis revealed that differences in liver fluxes and dietary cholesterol absorption could explain this occurrence of the two different phenotypes. In individual mice with dyslipidemia dietary cholesterol absorption and hepatic turnover of metabolites, including lipid fluxes, were higher compared to those without dyslipidemia. Predicted differences were also observed in gene expression data, and consistent with the emergence of insulin resistance and hepatic steatosis, two well-known MetS co-morbidities. Whereas MINGLeD specifically models the metabolic derangements underlying MetS, the simulation method ADAPT is generic and can be applied to other diseases where dynamic modeling and longitudinal data are available.

## Author summary

Obesity is becoming a growing health problem, with the risk of running into a disease state called the Metabolic Syndrome (MetS). It is difficult to study MetS in humans as it develops over a long period of time while many processes and organs are involved. Moreover, each patient may present itself with different characteristics. We combined data from animal experiments with a computer model and computer simulations to study the development of MetS. The new model correctly predicts which changes in the underlying metabolic processes could explain the MetS symptoms. Unexpectedly, two different subgroups were identified: those with high cholesterol and high triglycerides, and those without. The computer model found that in those who develop lipid abnormalities, both dietary cholesterol absorption and hepatic liver fluxes were higher.

## Introduction

The simultaneous presentation of obesity, dyslipidemia, insulin resistance and hypertension is generally referred to as the Metabolic Syndrome (MetS) [1–5]. Together, these factors impose increased risk for the development of co-morbidities including cardiovascular disease, type 2 diabetes and non-alcoholic fatty liver disease [6,7]. MetS is considered to be the result of an imbalance in the mechanisms controlling dietary intake, energy expenditure, glucose handling and lipid homeostasis [8–10]. The high prevalence of obesity and MetS [11–15], in combination with the heterogeneous presentation of MetS patients [16,17], asks for the design of adequate treatment and prevention strategies. Clinical research on MetS is mostly cross-sectional in nature and tends to focus on either lipid or glucose metabolism, while both make up MetS. Knowledge on the interplay between these different metabolic components during the relatively slow progression into disease is therefore limited, but which a systems biology approach could provide.

Modeling efforts in the past have shown that systems biology can be a powerful approach to gain both qualitative and quantitative insight in the inherently complex systems that drive the development of MetS. For example, Lu *et al*. [18] demonstrated how HDL raising modulators fail to reduce cardiovascular disease by elucidating the effects on the reverse cholesterol transport pathway. Likewise, Topp *et al*. [19] have identified different response pathways and physiological outcomes during prolonged hyperglycemia.

However, these computational models were designed to model the healthy [18,20–29] or diseased state exclusively [19,30]. Furthermore, these models only describe short-term dynamics (e.g. the postprandial response period) and do not take into account long-term dynamics that may be expected to occur in progressive diseases such as MetS. Moreover, models that do explicitly study the gradual phenotype transition into a diseased state, have only considered either the lipid component [31] or the glucose component [32–35] of MetS.

In addition, a long-term simulation method has been developed referred to as Analysis of Dynamic Adaptations in Parameter Trajectories (ADAPT) [36–38]. It infers time-varying parameters that gradually change over time, reflecting the slow change in regulation of metabolic processes during disease development. Therefore, ADAPT is a very powerful approach to study longitudinal development of diseases and therapeutic interventions and uses experimental data to infer adaptations in the system [36,37,39,40]. In previous studies, ADAPT has been applied to study hepatic steatosis [37,39], and treatment of type 2 diabetes [40], but has not yet been applied to study the full metabolic complexity of MetS.

Therefore, we aimed to design a computational, data-driven approach to study the longitudinal and progressive dynamics of the majority of metabolic alterations of MetS, i.e. obesity, glucose intolerance, insulin resistance and dyslipidemia. We employed a systems biology methodology that integrates three main concepts to infer metabolic adaptations during MetS development: i) the long-term simulation method ADAPT, combined with ii) a newly developed *in silico* MetS model that describes the metabolic processes involved in whole-body carbohydrate and lipid metabolism, and integrated with iii) time-series data obtained from an *in vivo* MetS model. The APOE*3-Leiden(E3L).CETP mouse [41,42] is a mouse model for human MetS that develops diet-induced dyslipidemia and is prone to develop obesity and insulin resistance. It has been used in cross-sectional studies addressing different metabolic facets of MetS [41,43–51]. We made use of this model to study the development of diet-induced MetS in a longitudinal setting [48,52] by collecting measurements in the same animals within a three-month period and at multiple intermediate time points.

Our *in silico* modeling quantitatively analyzes and integrates the experimental data and provides estimates of metabolite concentrations and fluxes that were experimentally not measured. With this modeling approach, we demonstrate when and how the onset and development of MetS occurs. Although we expected to find a homogeneous population, our modeling approach shows the emergence of different phenotypes of MetS. This heterogeneity is associated with differences in intestinal and hepatic metabolic fluxes.

## Results

We combined data-driven and physiology-based modeling. Hereto we integrated prior knowledge on the complex metabolic systems that underlie the pathophysiology of MetS with experimental observations on the actual metabolic status over time: *in vivo* data of the onset and progressive development of MetS have been collected from 11-week-old male E3L.CETP mice that were fed diets differing in fat and cholesterol content and were followed for three months. These diets comprised of a low-fat diet (LFD; 20% of energy from fat; n = 8), high-fat diet (HFD; 60% of energy from fat; n = 12) and a high-fat diet with additional dietary cholesterol (HFD+C; 0.25% cholesterol; n = 8). For each diet group, longitudinal data was obtained throughout the time course of the study: body weight was monitored weekly, plasma markers were measured monthly and liver lipids were assessed after three months. S1 Note provides a detailed overview of the experimental set-up and measurement details.

### Male E3L.CETP mice on a high-fat diet with cholesterol develop obesity, glucose intolerance and dyslipidemia

**Fig 1:**
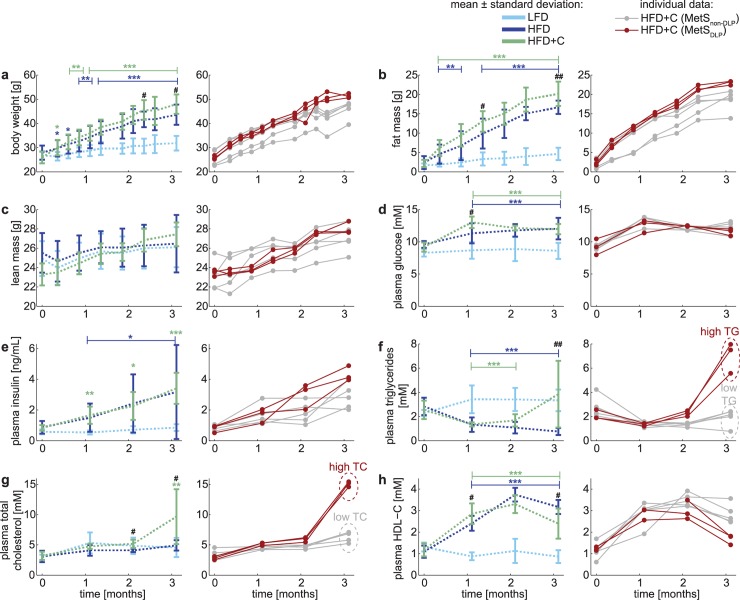
*In vivo* development of the Metabolic Syndrome results in different phenotypes. Experimentally observed metabolic parameters upon dietary induction in male E3L.CETP mice over the time course of three months is displayed in two ways: in the left panels the data are expressed as mean ± standard deviation (error bars) for the low-fat diet (LFD; n = 8; light blue), high-fat diet (HFD; n = 12 (pooled from two groups n = 7 for the full time period, n = 5 until 2 months of dietary induction; dark blue) and high-fat diet with 0.25% cholesterol (HFD+C; n = 8; green) groups, whereas in the right panels the data of the animals on HFD+C are depicted for each animal individually. Individuals in this cohort were subdivided into two groups based on the plasma triglyceride (TG) and plasma total cholesterol (TC) levels. The dyslipidemic Metabolic Syndrome phenotypes are depicted in red (MetS_DLP_; mice with high plasma TG and simultaneous high plasma TC at t = 3 months) and the non-dyslipidemic Metabolic Syndrome phenotypes in gray (MetS_non-DLP_; mice with low plasma TG and simultaneous low plasma TC at t = 3 months). Differences between groups were determined using one-way ANOVA test. When significant differences were found, Fisher’s LSD test was used as a post hoc test to determine the differences between two independent groups: * P<0.05; ** P<0.01; *** P<0.001 HFD as compared to LFD ^#^ P<0.05; ^##^ P<0.01; ^###^ P<0.001 HFD+C as compared to HFD.

Fig 1 presents the results of feeding LFD, HFD and HFD+C as average (left) and individually (right) for HFD+C feeding; the latter will be discussed in the next paragraph. All individual datasets are also available in S1 Data. As compared with LFD feeding, increased fat intake (HFD) resulted in increased weight gain (Fig 1A) reflected by a marked increase in fat mass (Fig 1B) with no changes in lean mass (Fig 1C). HFD feeding also significantly increased fasting plasma glucose (Fig 1D) and insulin (Fig 1E) levels. Although HFD did not affect the plasma total cholesterol level (TC; Fig 1G), a shift in the lipoprotein ratio was observed, reflected by increased plasma HDL-cholesterol (HDL-C) levels (Fig 1H) upon feeding a high-fat diet.

As compared to the HFD alone, obesity development was slightly increased by cholesterol feeding (Fig 1A and 1B), as monitored by increased fat mass at three months after starting the diet, even though the daily food intake did not increase (data not shown). Although the additional dietary cholesterol did not affect plasma glucose and insulin levels (Fig 1D and 1E), it did impair glucose tolerance in the oral glucose tolerance test (see S1 Note–Fig 1), indicating that dietary cholesterol increased insulin resistance.

As compared to HFD, plasma triglyceride (TG) levels were significantly increased after three months of feeding HFD with cholesterol (Fig 1F). Cholesterol feeding did not seem to have an effect on circulating TC levels until the second month of the dietary induction (Fig 1G). Plasma HDL-C level (Fig 1H), on the other hand, was increased after starting the diet (HFD and HFD+C) over the first two months.

### Male E3L.CETP mice respond bimodally to cholesterol feeding suggesting the presence of two distinct Metabolic Syndrome phenotypes

Male E3L.CETP mice developed HFD-induced obesity, glucose intolerance and dyslipidemia, mimicking the classical symptoms seen in human MetS. However, the high variability in both triglyceride and cholesterol levels complicated the interpretation of the dyslipidemic component. Therefore, we also presented the data of mice fed with the HFD+C individually in the right-hand side panels of Fig 1. When inspecting the levels of plasma TG (Fig 1F) and plasma TC (Fig 1G) at the three months’ time point, the data reveal clear differences among the individual mice: while some develop dyslipidemia upon feeding HFD+C (shown in red), others do not (shown in grey), suggesting a bimodal distribution. With this insight, we divided the HFD+C cohort into two subgroups: the mode with high plasma TG and high TC levels after three months of HFD+C is referred to as the dyslipidemic Metabolic Syndrome phenotypes (MetS_DLP_; n = 3) and the other mode as the non-dyslipidemic Metabolic Syndrome phenotypes (MetS_non-DLP_; n = 5). This subdivision shows a consistent pattern in both TG and TC at the three months’ time point, but is already present earlier in time. In fact, the onset of dyslipidemia is already noticeable after two months of the diet, and progresses further towards the three months’ time period. Note that the subdivision in phenotype development is a result of the HFD+C diet; no baseline differences are observed (t = 0 months in this data set), and these observations are limited to the HFD+C group and not present in HFD.

This separation into two phenotypes is clearly present in the lipid trait, but it is also reflected in markers of carbohydrate metabolism. Although the individual mice cannot be separated based on the fasting plasma glucose (Fig 1D) data, the fasting plasma insulin (Fig 1E) indicates that the MetS_DLP_ individuals are significantly more insulin resistant than the MetS_non-DLP_ individuals after at the three months’ time point. This trend is even more profound in the insulin dynamics in response to a glucose challenge test (S1 Note-Fig 1): MetS_DLP_ individuals show a markedly higher insulin peak that also lasts longer than the MetS_non-DLP_ individuals. This indicates that dyslipidemia and glucose intolerance develop in parallel, but it is unclear how these are causally related.

### MINGLeD describes metabolic snapshots accurately

**Fig 2:**
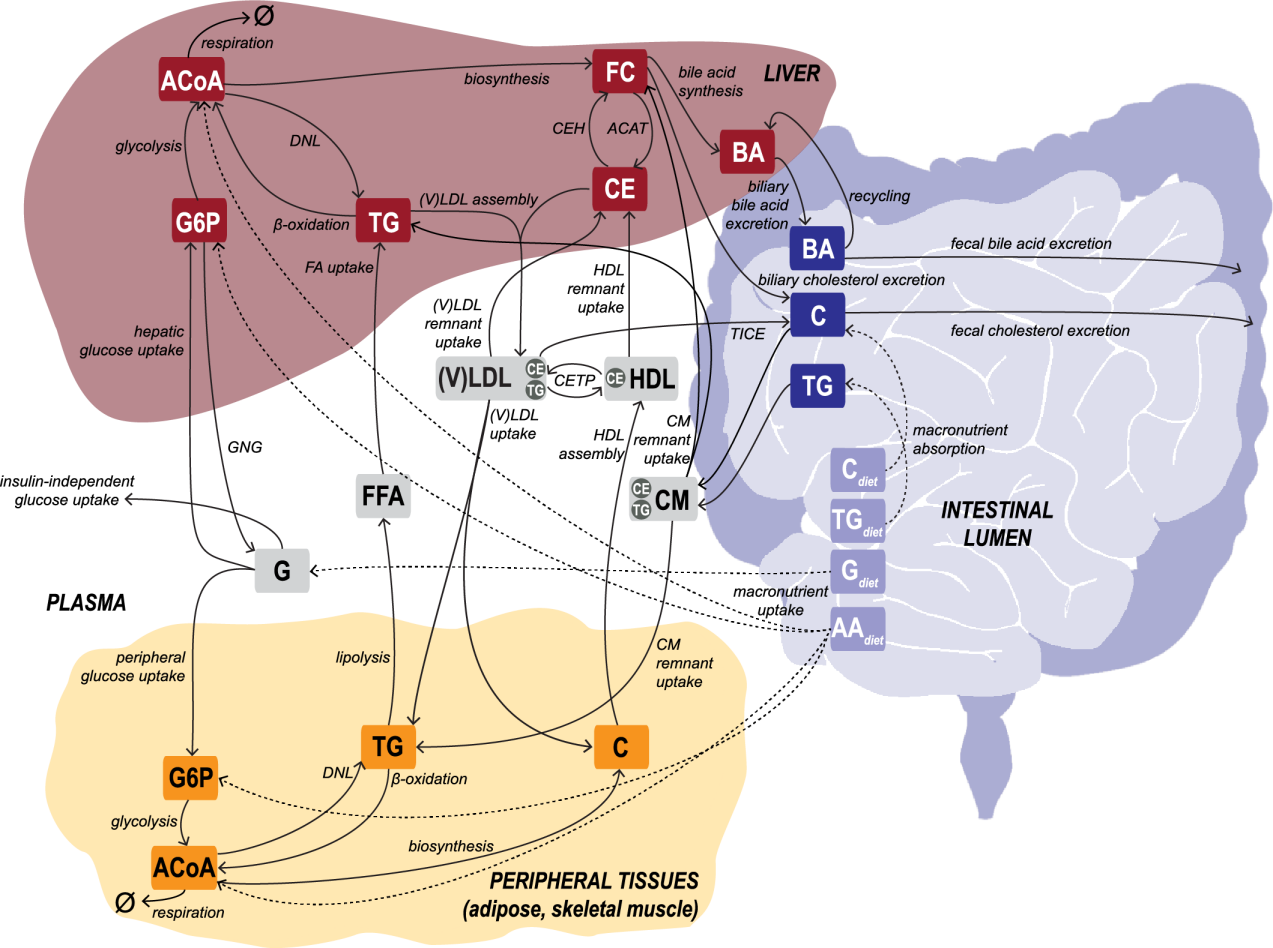
Schematic representation of the computational model MINGLeD. MINGLeD describes the metabolic pathways of glucose and lipids to describe the development of MetS. This multi-compartment framework encompasses pathways in dietary absorption, hepatic, peripheral and intestinal lipid metabolism, hepatic and plasma lipoprotein metabolism and plasma, hepatic and peripheral carbohydrate metabolism. The metabolite pools in the different tissue compartments are displayed in the frames; the corresponding metabolic fluxes are represented using the arrows. The dashed arrows represent the dietary inflow in terms of the different macronutrients derived from the experimental data. AA, amino acid; ACAT, Acyl-coenzyme A:cholesterol acyltransferase; ACoA, Acetyl CoA; BA, bile acid; C, cholesterol; CE, cholesteryl ester; CEH, cholesterol ester hydrolase; CETP, cholesteryl ester transfer protein; CM, chylomicron; DNL, *de novo* lipogenesis; (F)C, (free) cholesterol; (F)FA, (free) fatty acid; G, glucose; G6P, glucose-6-phosphate; GNG, gluconeogenesis; HDL, high density lipoprotein; TG, triglyceride; TICE, transintestinal cholesterol absorption; (V)LDL, (very) low density lipoprotein.

Next, we developed a novel *in silico* model that is tailored to describe data from longitudinal studies and simulates the metabolic system governing carbohydrate, lipid and cholesterol metabolism, providing predictions for unobserved fluxes and metabolites. The Model INtegrating Glucose and Lipid Dynamics (MINGLeD) is composed of a system of coupled, nonlinear ordinary differential equations (ODEs). The steady state of the ODE model represents a snapshot of the metabolic state and describes the mass balance of the metabolite pools and flux rates for carbohydrate, lipid and cholesterol species in the plasma, liver, intestinal lumen and periphery (Fig 2). The model aims to describe the metabolic system from a whole-body perspective under healthy conditions as well as at different stages during MetS development. All simulation code and *in silico* data files are available on GitHub (via https://github.com/yvonnerozendaal/MINGLeD).

**Fig 3:**
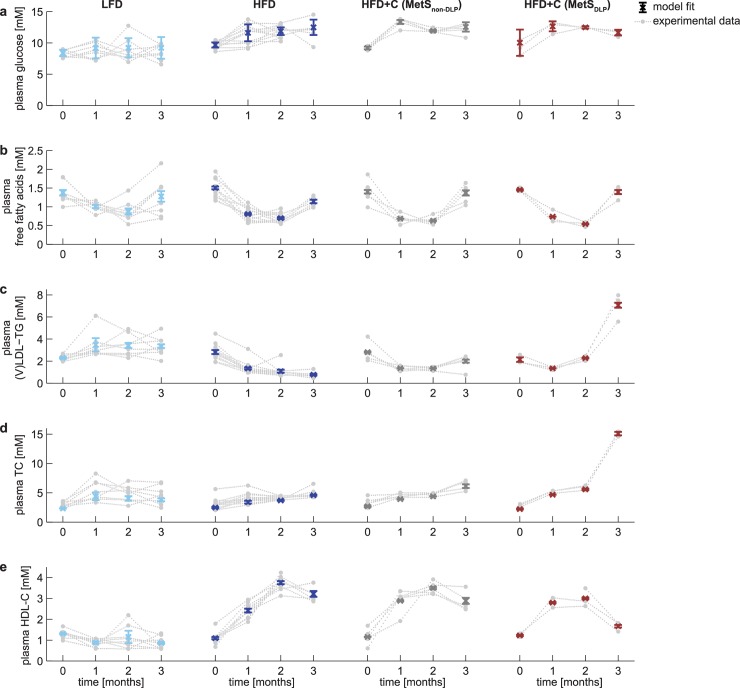
MINGLeD describes metabolic phenotypes of male E3L.CETP mice upon different diets and time points. The metabolic phenotypes are depicted for three different diets (with HFD+C composed of two subgroups that emerged after two months of dietary induction) at four different time points. Model fits (colored error bars: mean ± standard deviation) of MINGLeD calibrated to the phenotype snapshots (raw, individual mouse data shown in gray) separately. Only acceptable model simulations were included, which was classified as having a weighted sum of squared errors (see Eq 1 in S3 Note) below 100.

MINGLeD was integrated with the *in vivo* data whilst considering four subgroups: the LFD group, HFD group, MetS_non-DLP_ phenotypes and MetS_DLP_ phenotypes. For each subgroup, MINGLeD was fitted to the data of each of the four time snapshots separately. The resulting sixteen models differ in the values for their estimated parameters. For each model the parameter estimation procedure was repeated with 500 different initial parameter sets using multi-start optimization. Fig 3 displays that MINGLeD accurately fits the metabolic snapshots over time for each of these groups. This shows that MINGLeD is capable of describing different metabolic phenotypes upon varying dietary intake and at different metabolic stages in time.

### MINGLeD with ADAPT describes dyslipidemic and non-dyslipidemic Metabolic Syndrome progression

Calibration of MINGLeD yielded separate models for each subgroup (LFD, HFD, MetS_non-DLP_ and MetS_DLP_). However, this ignores the fact that the phenotypes represented by those four models are causally connected in time. MINGLeD describes metabolic fluxes and concentrations but does not explicitly include the multiple pathways that regulate and modulate metabolism over a three-month time period (such as changes in gene expression and protein activity). Both limitations were overcome by combining MINGLeD with a dedicated approach for longitudinal modeling of biological systems: ‘Analysis of Dynamic Adaptations of Parameter Trajectories’ (ADAPT) [36–38]. ADAPT uses the experimental data to infer adaptations in the system, which is implemented by introducing time-varying model parameters. Model parameters are iteratively re-estimated over the time course of the simulation, yielding parameter trajectories that govern the time-dependent evolution of the modeled state variables and fluxes. Combining MINGLeD with ADAPT and the experimental data resulted in a dynamic, continuous model of MetS development and progression. ADAPT uses ensemble-based simulation to account for both methodological and experimental uncertainty resulting from biological variability and relatively low power in the HFD+C subgroups. By repeated Monte Carlo sampling of the *in vivo* data, sampled datasets are generated and for each sampled dataset a parameter trajectory was estimated. This was repeated resulting in a set of 1,000 simulated models yielding a database comprising of *in silico* populations of 1,000 virtual individuals for each subgroup. Each simulation describes the experimental data adequately, though with variation in parameter trajectories yielding differences in fluxes and concentrations, especially for model variables that are not experimentally observed. A more detailed explanation of the ADAPT methodology is given in the Online Methods.

**Fig 4:**
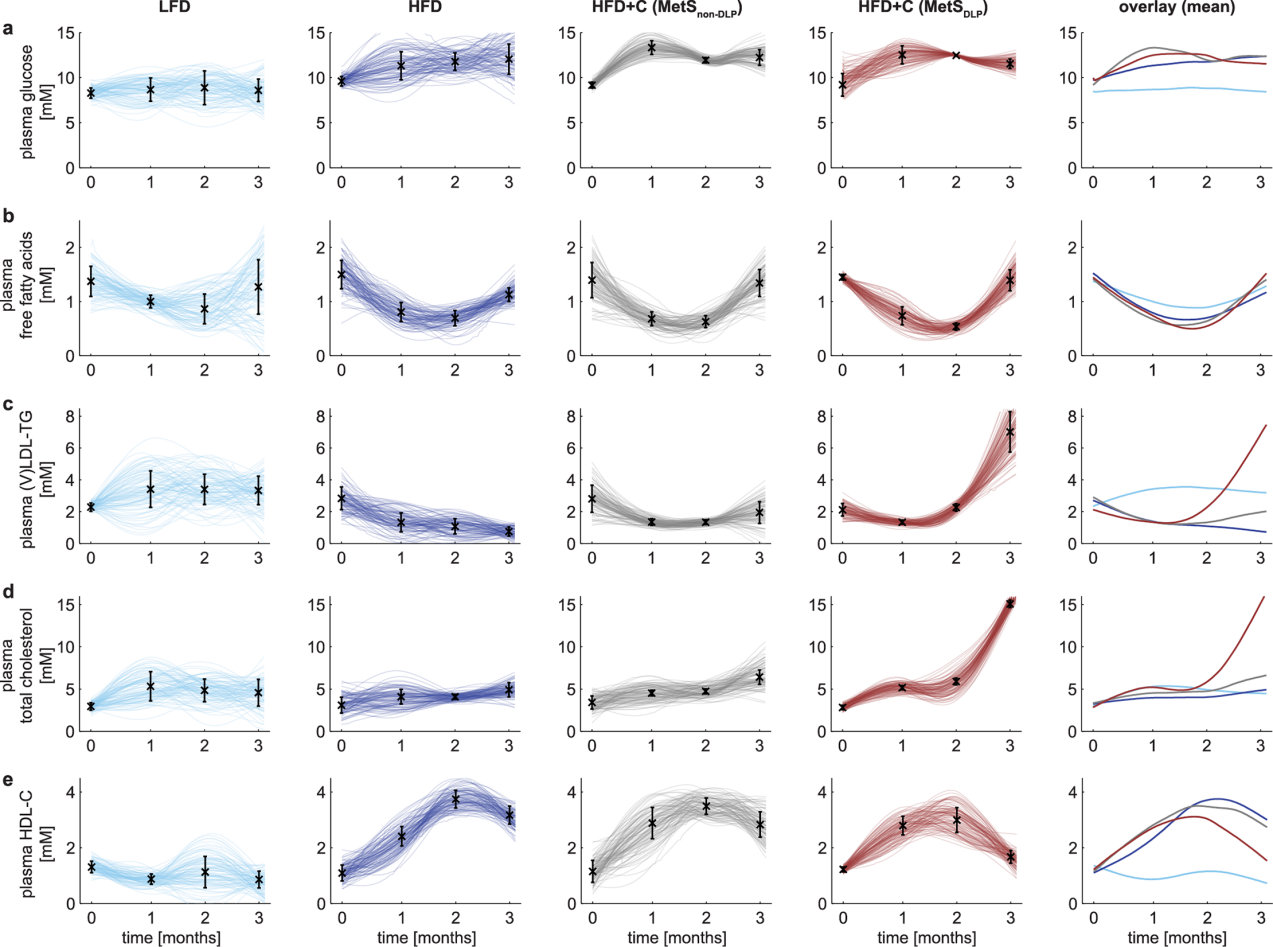
Onset and development of the Metabolic Syndrome reveals two distinct phenotypes of dyslipidemic status. The classical hallmarks of MetS are depicted by simulations of the individual trajectories (the top 10% best were selected from n = 1,000) for the low-fat diet (LFD) are shown in light blue; high-fat diet (HFD) in dark blue, the non-dyslipidemic Metabolic Syndrome phenotypes (MetS_non-DLP_) in gray and the dyslipidemic Metabolic Syndrome phenotypes (MetS_DLP_) in red. The color intensity reflects the density of the trajectories: the darker, the more probable the simulated solution. Experimental *in vivo* data are shown as black error bars that represent mean ± standard deviation. The 5^th^ column shows an overlay of the mean of the trajectories for each of the subgroups showing the development of increased triglycerides and cholesterol levels in the plasma in the dyslipidemic MetS phenotypes between two and three months.

Fig 4 shows that the simulated trajectories for the plasma metabolites fit the *in vivo* data points accurately and provide a continuous description of the dynamics with which the system behaves over time, illustrating the different trajectories for the different subgroups. Note that ADAPT did not yield one single simulation but provided ensembles of state and flux trajectories. Each line in Fig 4 is one trajectory solution, where a darker color represents more overlap (higher density) of trajectory solutions in that region. These density plots cover the solution space for each of the modeled components. To aid visual analysis, the mean of the trajectory distributions for each of the subgroups is depicted in the panels to the far-right. These trajectories illustrate the clear distinction that can be made between the dyslipidemic and non-dyslipidemic MetS phenotypes.

### Flux trajectory analysis identifies distinct differences in dietary cholesterol absorption and hepatic metabolism during dyslipidemia

**Fig 5:**
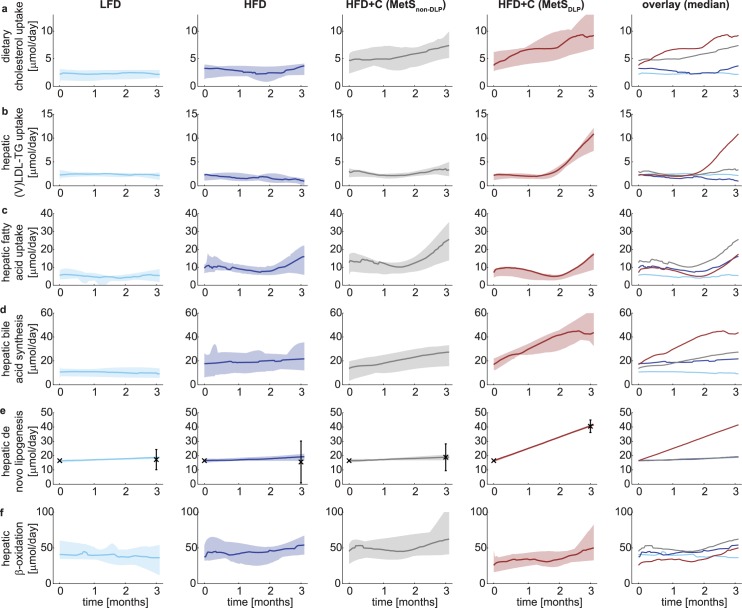
Metabolic flux trajectory analysis depicts differences among phenotypes and dyslipidemia development. Trajectory analysis reveals decreased dietary cholesterol absorption from the intestinal lumen in the non-dyslipidemic Metabolic Syndrome phenotype (a) and increased hepatic activity in the dyslipidemia Metabolic Syndrome phenotype (b-f). The median metabolic flux trajectories (calculated from the top 10% best trajectories from n = 1,000) are depicted with a solid line for the hepatic dietary cholesterol absorption from the intestinal lumen (a), hepatic (V)LDL-TG uptake from the plasma (b), hepatic fatty acid uptake from the plasma (c), hepatic bile acid synthesis from cholesterol (d), hepatic *de novo* lipogenesis (e), and hepatic β-oxidation (f). The shaded area depicts the 10% range of trajectories around the median. The low-fat diet cohort is depicted in light blue; the high-fat cohort in dark blue; the non-dyslipidemic Metabolic Syndrome phenotype in gray and the dyslipidemic Metabolic Syndrome phenotype in red. The experimental hepatic *de novo* lipogenesis (e) data are shown as black error bars that represent mean ± standard deviation.

We showed that the *in silico* model accurately captures the trends as observed in the *in vivo* data. The ensembles of simulated concentration and flux trajectories provide insight in how the underlying metabolic network is affected upon emergence of the two different MetS phenotypes. Fig 5 displays the median with 10% range of the collection of trajectories for several metabolic fluxes. Clear differences between the dyslipidemic and non-dyslipidemic MetS phenotypes can be observed. A complete overview of all model state trajectories and metabolic flux trajectories is documented in S1 Fig.

Despite equal intake of food and thus cholesterol, dietary cholesterol absorption from the intestinal lumen was markedly higher in the dyslipidemic compared to the non-dyslipidemic MetS group (Fig 5A), which may have promoted the development of dyslipidemia.

The modeling also predicted that within the dyslipidemic phenotype, rates of lipid shuttling towards (V)LDL-TG uptake (Fig 5B; see also S1K–S1L Fig) were increased, as higher rates of hepatic fatty acid uptake (Fig 5C) and lipogenesis (Fig 5E) were observed accompanied by a lower rate of hepatic β-oxidation (Fig 5F).

We observed similar changes if we analyze the gene expression data (S1 Note-Table 3) from the same animals: we could qualitatively relate our flux predictions with the gene expression profiles of the fully developed MetS phenotype after three months of dietary induction. We found, as compared to LFD, an upregulation of hepatic fatty acid uptake in the HFD, HFD+C (MetS_non-DLP_) and HFD+C (MetS_DLP_) groups (Fig 5C).

Fig 5D shows that hepatic bile acid synthesis is predicted to be higher in the MetS_DLP_ phenotype. This aspect could also be observed in the gene expression data: the expression of bile acid synthesis genes nuclear farnesoid X receptor (*FXR*) and Cholesterol 7 alpha-hydroxylase (*Cyp7a1*) was largely upregulated, as compared to HFD alone.

Hepatic *de novo* lipogenesis (Fig 5E; DNL) is predicted to be higher in the dyslipidemic phenotype, for which data at the 3 months’ time point was available and was used in the model fitting. Genes related to DNL, including Fatty Acid Synthase (*FASN*), Acetyl-CoA carboxylase 2 (*ACC2*), acyl-CoA:diacylglycerol acyltransferase 2 (*DGAT2*) and sterol regulatory element binding protein 1c (*SREBP1c*) were all largely downregulated probably as a compensatory mechanism.

In addition, the hepatic β-oxidation (Fig 5F) is predicted to be lower in the dyslipidemic group. This could be linked to a compensatory upregulation of genes related to fatty acid β-oxidation such as Peroxisome proliferator-activated receptor α (*PPARα*), Peroxisomal acyl-coenzyme A oxidase 1 (*ACOX1*) and Carnitine palmitoyltransferase 1a (*CPT1a*). Collectively, these predictions suggest more lipid accumulation in the liver of the dyslipidemic mice.

### Both Metabolic Syndrome phenotypes develop hepatic steatosis

**Fig 6:**
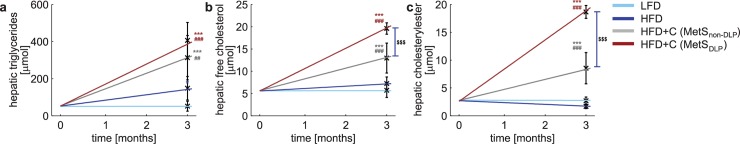
Metabolic Syndrome development is associated with hepatic steatosis in both dyslipidemic and non-dyslipidemic phenotypes. The mean trajectories of the liver lipid profiles (calculated from the top 10% best trajectories from n = 1,000) are depicted for the hepatic triglyceride pool (a), hepatic free cholesterol pool (b) and the hepatic cholesteryl ester pool (c). Experimental data was obtained at the end of the study and is depicted by the black error bars representing mean ± standard deviation for each of the groups. The data from the LFD cohort is used as initial value, assuming no hepatic lipid accumulation to have occurred in this control group. Differences between groups were determined using one-way ANOVA test. When significant differences were found, Fisher’s LSD test was used as a post hoc test to determine the differences between two independent groups: * P<0.05; ** P<0.01; *** P<0.001 as compared to LFD ^#^ P<0.05; ^##^ P<0.01; ^###^ P<0.001 as compared to HFD ^$^ P<0.05; ^$$^ P<0.01; ^$$$^ P<0.001 as compared to HFD+C (MetS_non-DLP_).

MINGLeD with ADAPT predicted the trajectories of the liver lipid profiles and calculated the hepatic lipid pool sizes over the entire time course of three months. Next, we measured lipid turnover and the lipid content in livers from individual mice after three months of dietary induction to verify these in *silico* predictions at the last time point. Indeed, addition of cholesterol did lead to an increase in lipid turnover and the hepatic lipid pool sizes as compared to the high-fat diet without cholesterol, both in the dyslipidemic and non-dyslipidemic phenotype (Fig 6). Moreover, this accumulation of lipids in the liver, in particular cholesterol components, was more profound in MetS_DLP_ mice.

Flux trajectory analysis revealed substantial differences in hepatic fluxes between the non-dyslipidemic and the dyslipidemic MetS phenotypes. In terms of plasma metabolite levels (see Fig 4A–4E), MetS_non-DLP_ mice closely resemble the mouse population fed the HFD without cholesterol, and intriguingly, appears not to be affected by the additional dietary cholesterol load. MINGLeD with ADAPT predicted that this may be due to reduced dietary cholesterol absorption (Fig 5A) in the non-dyslipidemic phenotype.

Furthermore, the predicted lipid pool sizes were in agreement with liver histology data (S1 Note-Fig 2), which showed the establishment of microvesicular steatosis upon HFD feeding. In contrast, steatosis was exacerbated in MetS_DLP_ mice revealed by a more severe type of macrovesicular steatosis.

## Discussion

Our goal was to design an approach to study the longitudinal and progressive dynamics of metabolic alterations of MetS. Not only do the results show that our systems biology approach successfully describes the multitude of the metabolic changes, our modeling approach also describes the development of clinically relevant pathophysiological symptoms, including liver lipid accumulation (hepatic steatosis) and reduced insulin sensitivity (pre-diabetes). This demonstrates that our modeling approach can be used to study the onset and progression of MetS as well as its co-morbidities. From the heterogeneous dataset our model identified differences among tested individual mice. Unexpectedly, the existence of two different phenotypes in MetS development was predicted.

### Heterogeneity in phenotype development

The clinical presentation of MetS in humans is highly heterogeneous and spans over decades. Male E3L.CETP mice fed a high-fat diet supplemented with cholesterol develop MetS within a time scale of several months. Although all of these animals have the same genetic background, received the same diet and were kept and monitored in a controlled, standardized environment, this *in vivo* model did show heterogeneity in phenotypic presentation. In addition, the manifestation of the full repertoire of metabolic alterations associated with MetS makes this a useful *in vivo* model, whereas other animal models only describe one or partial metabolic aspects of MetS [53–59].

Using a traditional statistical approach, both this heterogeneity and limited datasets comprising of low number of animals are problematic. Moreover, the time-dependency of the data–i.e. individual data of consecutive points in time are interrelated and therefore not independent samples–would further complicate analysis. Our computational modeling approach tackles these problems by combining MINGLeD with ADAPT. Contrary to other computational models, MINGLeD integrates both glucose and lipid species at a whole-body level. Both carbohydrates and fats are of importance in MetS and MINGLeD allows for simultaneous description of these key components in terms of both metabolite pool sizes (concentrations) and metabolic fluxes. Complexity and detail in model equations was considered in close relation to what is experimentally feasible to measure throughout long-term MetS development. Therefore, MINGLeD’s data-driven, physiological design allows for describing both flux and concentration data on a whole-body level. MINGLeD per se can be simulated to describe metabolic snapshots, whereas the long-term dynamics are captured by using MINGLeD in conjunction with ADAPT. ADAPT has been designed to work with this kind of data and makes use of the time-dependent observations and simultaneously assesses uncertainty based on the variability in the data. The strength of the ADAPT methodology in dealing with heterogeneity became evident with the identification of two distinctly different phenotypes despite the limited number of animals that were studied. These two phenotypes mainly differ in terms of dyslipidemia, and flux trajectory analysis pinpointed differences in: 1) hepatic turnover of metabolites, including lipid fluxes and 2) the intestinal cholesterol absorption. This appears to mimic the observation in humans showing that levels of cholesterol absorption efficiency can vary greatly among individuals [60,61]. These model predictions are open for further experimental validation.

### Future perspectives

The methodology of integrating *in vivo* and *in silico* information allows to combine pre-existing knowledge with experimental quantitative data and can therefore be applied to study other multifactorial, progressive diseases where longitudinal data are available. A future application of the model is to quantify energy intake and energy expenditure and analyze the energy balance over time for development of obesity and MetS.

### Conclusion

In conclusion, we combined data from animal experiments with a computer model and computer simulations to study the development of MetS. The new model predicted which changes in the underlying metabolic processes could explain the MetS symptoms. Two different subgroups were identified: those with high cholesterol and high triglycerides, and those without. The computer model found that in those who develop lipid abnormalities, both dietary cholesterol absorption and hepatic liver fluxes were higher.

## Materials and methods

### Ethics statement

All animal experiments were performed in accordance with the regulations of Dutch law on animal welfare, and the Animal Ethics Committee of the Leiden University Medical Center, Leiden, The Netherlands. Animals were sacrificed by CO_2_ inhalation.

### *In vivo* model of Metabolic Syndrome development

The first step of our systems approach involves the gathering of *in vivo* data at different stages during the development of the Metabolic Syndrome. To this end we use male APOE*3-Leiden(E3L).CETP transgenic mice as diet-induced *in vivo* model to study the metabolic adaptations that occur over time.

Male E3L.CETP transgenic mice were housed under standard conditions with a 12 h light/dark cycle (7AM-7PM), housed with 1–2 animals per conventional cage with free access to chow diet and water, unless indicated otherwise. At the age of 11 weeks, randomized according to body weight and plasma lipids (total cholesterol and triglycerides) and glucose, mice were divided into three groups: mice were fed either a low-fat diet (LFD; n = 8), high-fat diet (HFD; n = 12) or a HFD with additional cholesterol (HFD+C; 0.25%, w/w; Sigma) (HFD+C; n = 8) for three months. The LFD has a 20% energy content derived from lard and contains 3.8 kcal/g diet; the HFDs have a 60% energy content derived from lard and contain 5.2 kcal/g diet (OpenSource Diets, Research Diets, Inc. New Brunswick, USA). The specific composition of each diet is listed in S1 Note-Table 1.

During the study, body weight and food intake were measured weekly, body composition (lean and fat mass) every other week. Blood samples were taken monthly and analyzed for glucose, insulin, free fatty acids, total cholesterol, HDL-cholesterol and triglycerides. At the end of the three months dietary induction experiment, animals were sacrificed. Livers were isolated for measuring lipid metabolites and gene expression, and the hepatic *de novo* lipogenesis was determined by using an isotope tracer. For further details on the experimental setup the reader is referred to S1 Note. All individual data is available as S1 Data.

### *In silico* model describing Metabolic Syndrome development

The second step of our systems approach involves the development of a dedicated *in silico* model describing MetS onset and progression. To this end we have developed a novel dynamic, computational model called Model INtegrating Glucose and Lipid Dynamics (MINGLeD). This model is tailored to describe data from longitudinal studies and involves the metabolic system governing carbohydrate, lipid and cholesterol metabolism. It is composed of a system of coupled, nonlinear ordinary differential equations (ODEs). The steady state of the ODE model represents a snapshot of the metabolic state and describes the mass balance of the metabolite pools and flux rates for carbohydrate, lipid and cholesterol species in the plasma, liver, intestinal lumen and periphery. This *in silico* model aims to describe the metabolic system in a whole-body perspective under healthy conditions as well as at different stages during Metabolic Syndrome development.

The steady state of MINGLeD describes the average behavior of the metabolic system over the time course of one day. Therefore no specific postprandial or fasting periods have been included explicitly. The metabolic pathways that are important in describing the metabolic system are presented in Fig 2. The exact block diagram of the computational model is schematically represented in S2 Note-Fig 1. The system of ODEs and fluxes equations are derived in S2 Note and listed in S2 Note-Tables 1–2.

MINGLeD describes metabolite pools originating from carbohydrate substrates (glucose, glucose-6-phosphate, acetyl coenzyme a), lipid species (free fatty acids, triglycerides, various lipoproteins) and cholesterol (free cholesterol, cholesteryl esters and bile acids). The metabolic pathways that define the interactions between these metabolites include the uptake of dietary macronutrients, glycolysis, hepatic gluconeogenesis, lipoprotein assembly and (remnant) uptake, cholesteryl ester transfer between lipoproteins (upon action of the cholesterol ester transfer protein; CETP), trans-intestinal cholesterol excretion (TICE), exchange between free cholesterol and cholesteryl esters (via ACAT and CEH) in hepatic tissue, hepatic fatty acid uptake, peripheral lipolysis, β-oxidation, *de novo* lipogenesis (DNL) and cholesterol biosynthesis. Bile acid synthesis, biliary bile acid and cholesterol excretion, enterohepatic reuptake, fecal excretion of bile acids and cholesterol. Finally, metabolism of acetyl coenzyme A in hepatic and peripheral tissues has been included.

### Modeling metabolic snapshots of different phenotypes

To validate the proposed structure of the *in silico* model, we calibrate MINGLeD to the *in vivo* data of the different metabolic snapshots. The experimentally measured metabolites are closely related to many of the variables in the computational model, such that an identifiable model is achieved of which the model parameters can be determined using parameter estimation. For each of the diet cohorts, MINGLeD is fitted separately to the phenotype snapshot determined at each month during the dietary induction period. For each snapshot, we compute the average and standard deviation of the measured data at this time point and use this data to fit the model to using maximum likelihood estimation. S3 Note and S3 Note-Table 1 describe how we relate the experimentally observed data to the specific model outputs and equations using the weighted sum of squares as error measure to be minimized. A Monte Carlo approach is employed to account for methodological and experimental uncertainties. The optimization procedure is repeated 500 times using a widely dispersed range of initial parameter values (10^−1^–10^1^) to accommodate multi-start optimization. The implementation details can be found in S2 Note. Once optimized, MINGLeD predicts unobserved species and fluxes and is used to make predictions on the underlying differences between phenotype snapshots.

### Modeling phenotype transition: Modeling the onset and progressive behavior over time

The next step in our systems approach is to couple the phenotype snapshots in time. Hereto we make use of the computational technique entitled ‘Analysis of Dynamic Adaptations of Parameter Trajectories’ (ADAPT) [36–38]. By employing this concept of the time-dependent evolution of model parameters, dynamic disease trajectories are obtained from which the onset and progression of MetS symptoms and co-morbidities can be studied. The progression of these adaptations is predicted by identifying necessary dynamic changes in the model parameters to describe the transition between experimental data observed at different points in time during the dietary induction.

Since it is *a priori* unknown which model parameters change with time, it is not possible to perform a dynamic simulation of the entire time span in one go. Therefore we discretize the time span into 90 segments, each representing one day. ADAPT interpolates between the individual snapshots in time (at which experimental data was obtained) and simulates every day in between these time points. To facilitate this, some pre-processing steps are required. Since the quantitative experimental data is discrete and only available at four points in time, the data is interpolated using cubic smoothing splines to obtain continuous dynamic descriptions of the experimentally observed metabolite pools and fluxes. To account for experimental and biological uncertainties, a collection of 1,000 splines is calculated using a Monte Carlo approach: different random samples of the experimental data are generated assuming Gaussian distributions with means and standard deviations of the data. Subsequently, for each generated sample, a cubic smoothing spline is calculated. This bootstrapping approach yields samples of data replicates which will subsequently be utilized in parameter estimation [62]. By combining bootstrapping of data, sampling of parameters and a robust optimization of model simulations, ADAPT provides feedback about uncertainty in model predictions accounting for uncertainty in both experimental measurements and fitting procedures.

The system is first simulated for the phenotype prior to the dietary induction by optimization to the t = 0 data. For each step in time, the system is simulated using the final values of the model states of the previous time step as initial conditions. Since we consider the parameters to be time-varying, the model parameters are re-optimized for each time step by minimizing the difference between the (sampled and interpolated) experimental data and the corresponding model outputs. The estimated parameter set from the previous time point is used as initial set for the optimization procedure. It is assumed that the induced adaptations are minimal and proceed progressively in time. Therefore, highly fluctuating parameter trajectories are considered to be non-physiological. To prevent the occurrence of such behavior, the parameter estimation protocol is extended to prevent unnecessary change of parameters and to identify minimal parameter adaptations that are required to describe phenotype transition. The cost function is extended with a regularization term (see Eq 2 in S3 Note), given by the sum of squared derivatives of the normalized parameter values. Hence, changing a parameter is costly, and will therefore be avoided if this is not required to describe the experimental data. The constant λ that determines the strength of this regularization term, should be chosen carefully such that the data fitting is biased as little as possible. If too large a value for λ is chosen, the regularization term becomes dominant and the model will not describe the experimental data accurately anymore. Since a small λ is already sufficient to minimize parameter changes and fluctuations, whilst still describing the experimental data accurately, λ was set to 0.1 in this study.

All simulation code and *in silico* data files are available on GitHub (via https://github.com/yvonnerozendaal/MINGLeD).

ADAPT yields a collection of parameter sets that describe the dynamics of the onset and development of MetS over time. The obtained trajectories for the model parameters, but also for the fluxes and pool sizes provide insight in the affected underlying biological system. This provides information about the adaptations that have taken place during the dietary induction, and these model-based predictions are compared to the gene expression data that is measured at the end of the dietary induction study.

## Supporting information

S1 Fig Predicted metabolite pools and flux trajectories. Panels a-b display the dynamics in metabolite pools over time and panels e-t display the corresponding flux trajectories. We selected the n = 100 best trajectories (top 10% based on WSSE). The 10% range around the median trajectory is depicted by the shaded area and the median trajectory for each model component is depicted by the solid line for the low-fat diet group (light blue), high-fat diet group (dark blue), non-dyslipidemic Metabolic Syndrome phenotype (gray) and the dyslipidemic Metabolic Syndrome phenotype (red) respectively. Experimental data is represented by the black error bars (mean ± standard deviation).
(PDF)

S1 Note Experimental setup. (PDF)

S2 Note Detailed model description of MINGLeD. (PDF)

S3 Note Data used for model calibration. (PDF)

S1 Data Raw *in vivo* data file. (XLSX)
