# Supplementary material for: In vivo and in silico dynamics of the development of Metabolic Syndrome
Source: PLoS Comput Biol. 2018 Jun 7;14(6):e1006145. doi: 10.1371/journal.pcbi.1006145 (PMC5991635; doi:10.1371/journal.pcbi.1006145)
Supplement: S1 Fig — Panels a-b display the dynamics in metabolite pools over time and panels e-t display the corresponding flux trajectories. We selected the n = 100 best trajectories (top 10% based on WSSE). The 10% range around the median trajectory is depicted by the shaded area and the median trajectory for each model component is depicted by the solid line for the low-fat diet group (light blue), high-fat diet group (dark blue), non-dyslipidemic Metabolic Syndrome phenotype (gray) and the dyslipidemic Metabolic Syndrome phenotype (red) respectively. Experimental data is represented by the black error bars (mean ± standard deviation). (PDF) [file pcbi.1006145.s001.pdf]

S1 Figure: Predicted metabolite pools and flux trajectories

Supplemental material for "*In vivo* and *in silico* dynamics of the development of Metabolic Syndrome" by Y.J.W. Rozendaal, Y. Wang, Y. Paalvast, L.L. Tambyrajah, Z. Li, K. Willems van Dijk, P.C.N. Rensen, J.A. Kuivenhoven, A.K. Groen, P.A.J. Hilbers, N.A.W. van Riel

a – Plasma metabolite pools

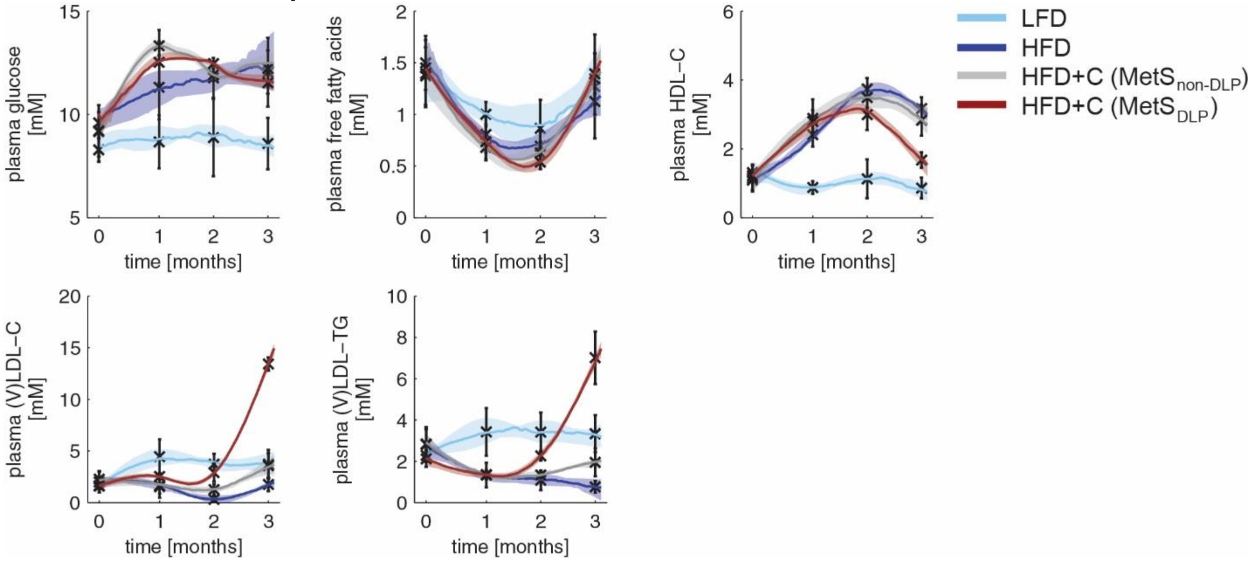

b – Hepatic metabolite pools

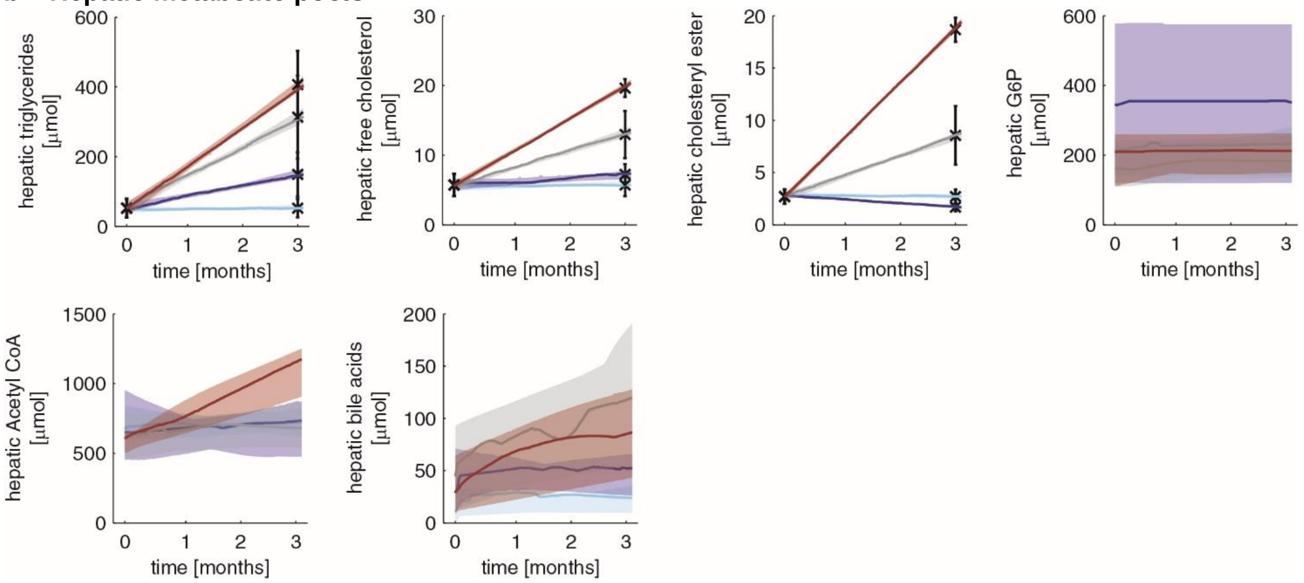

c – Peripheral metabolite pools

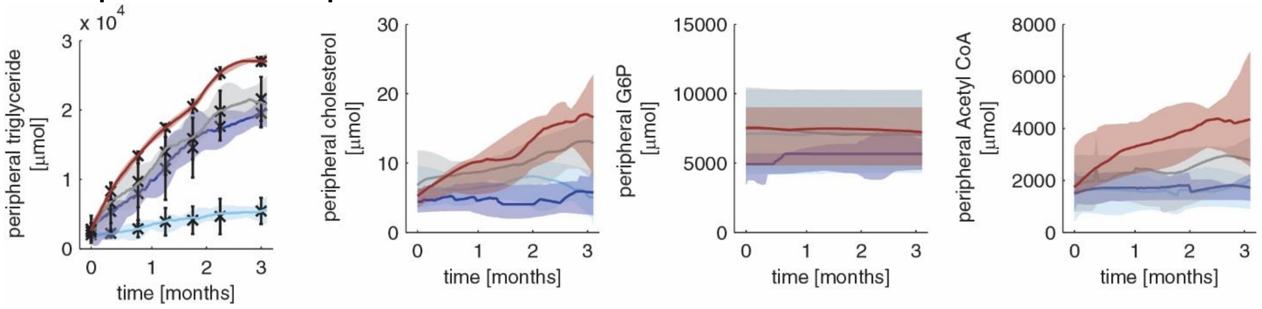

d – Metabolite pools in the intestinal lumen

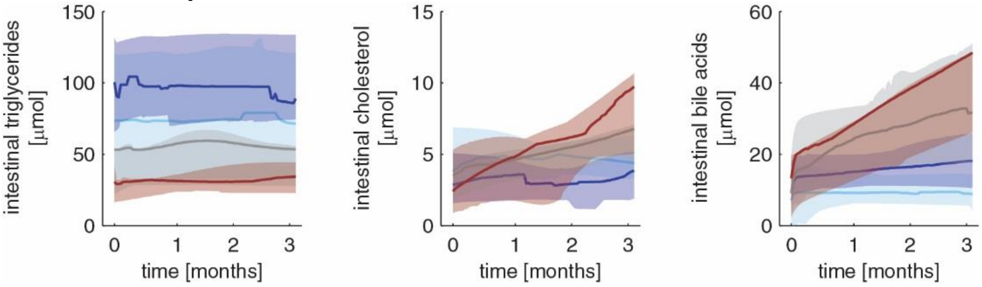

e – Dietary intake in terms of macronutrients

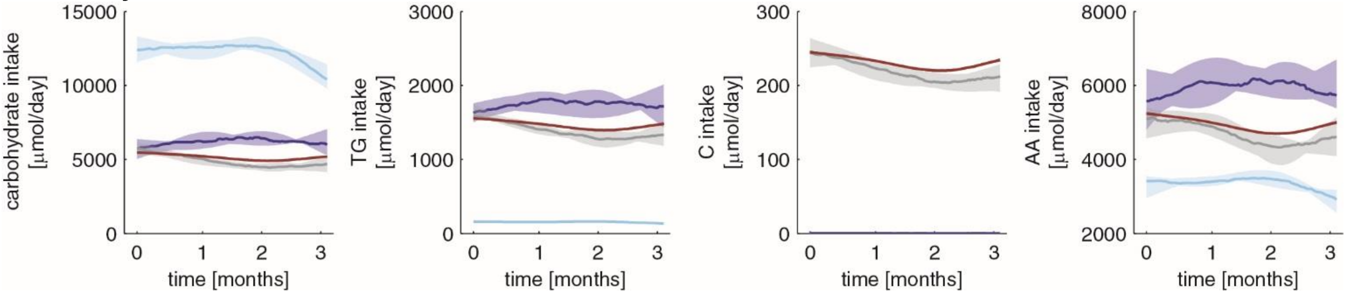

f – Dietary triglyceride and cholesterol uptake

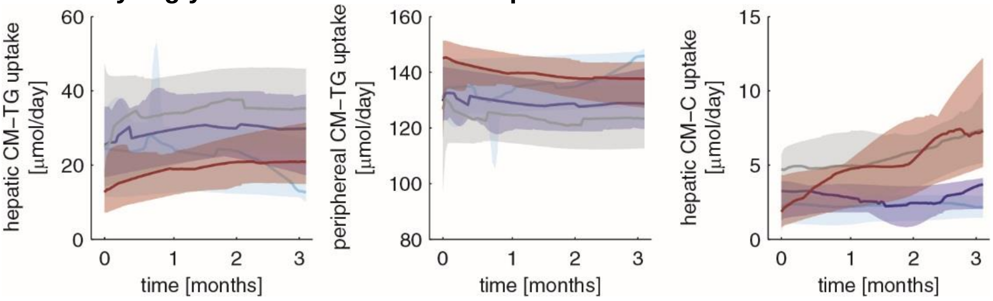

g – Dietary amino acid uptake fluxes

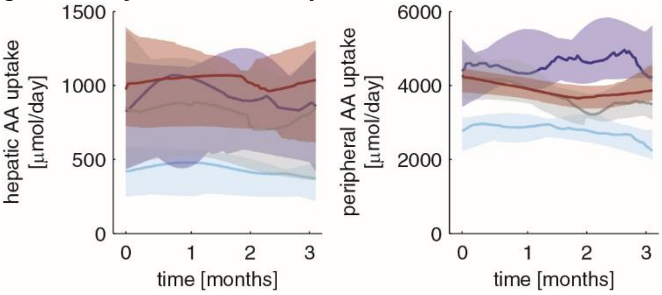

h – Amino acid uptake via the glucogenic (50%) and ketogenic (50%) pathway in liver and periphery

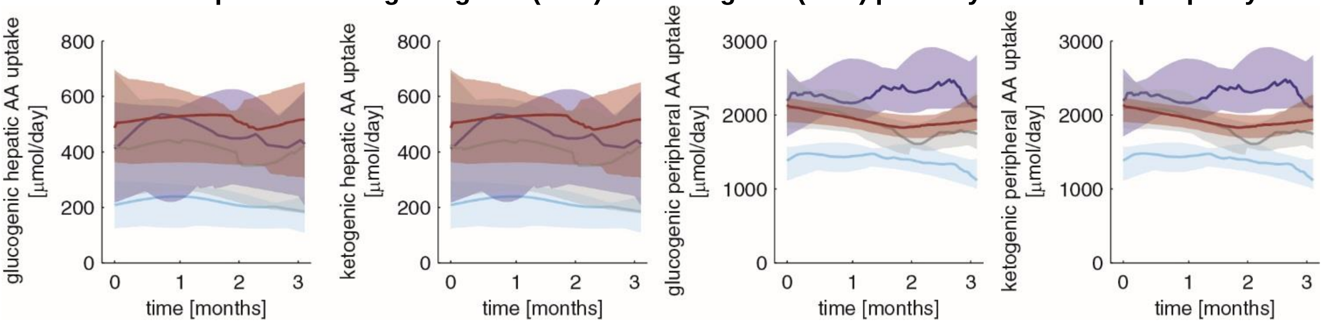

i – Dietary carbohydrate uptake fluxes

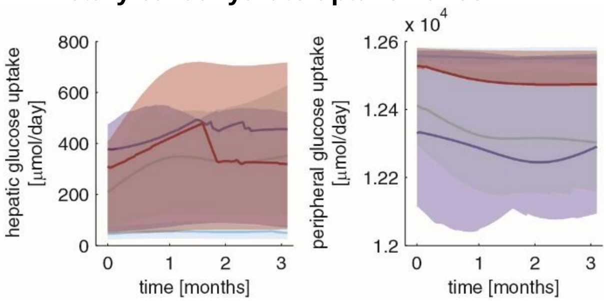

j – Carbohydrate metabolic fluxes

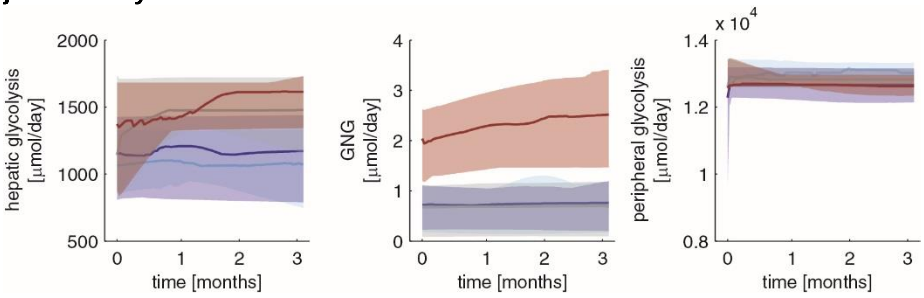

k – Lipoprotein formation fluxes

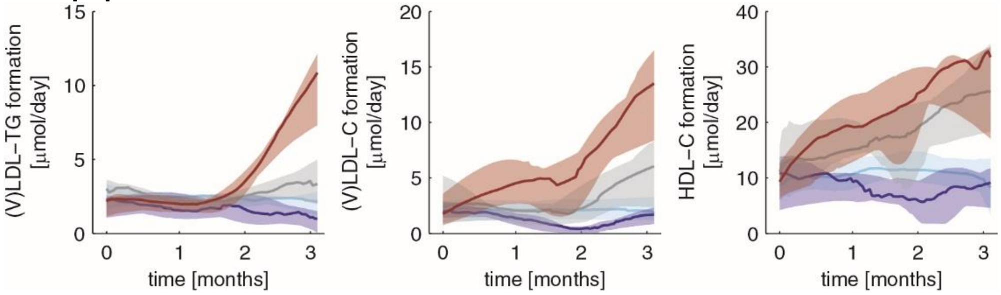

l – Lipoprotein (remnant) uptake fluxes

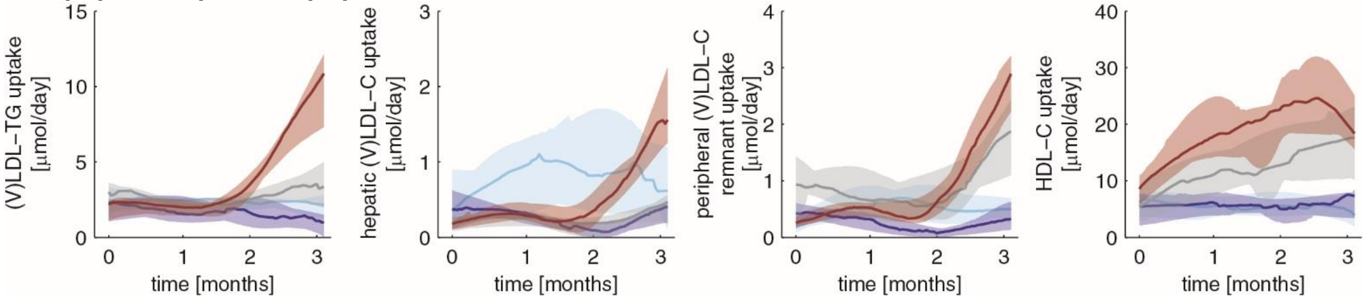

m – Lipoprotein metabolism fluxes

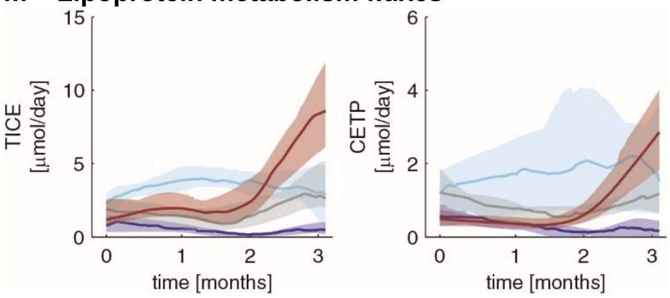

n – Fatty acid uptake flux

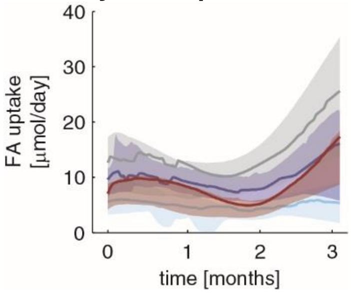

o – Hepatic metabolic fluxes

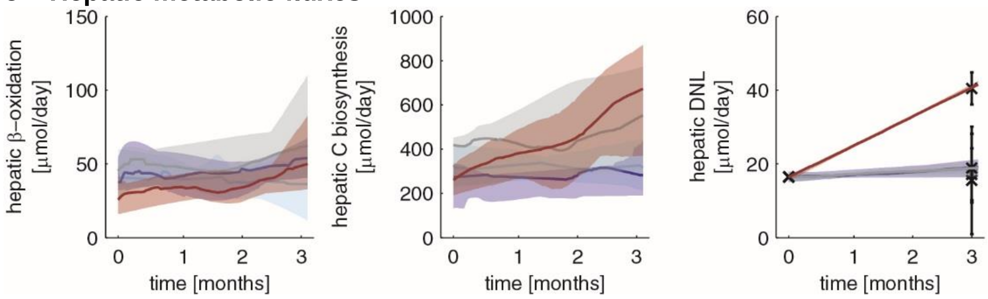

p – Peripheral metabolic fluxes

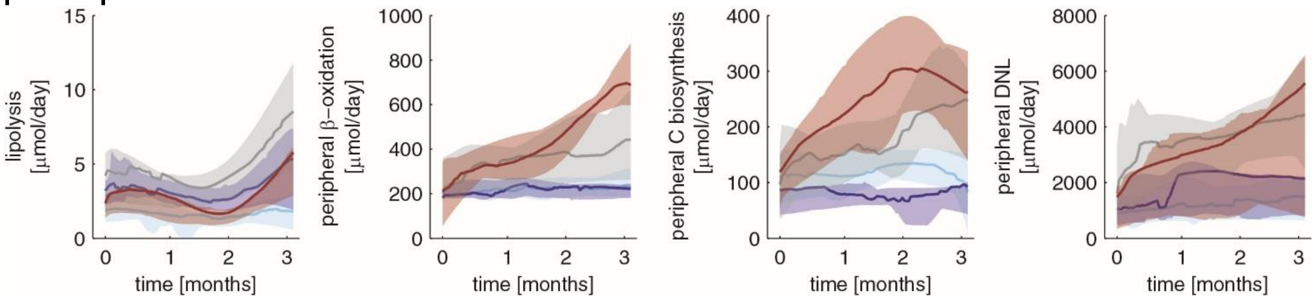

q – Hepatic cholesterol storage and release fluxes

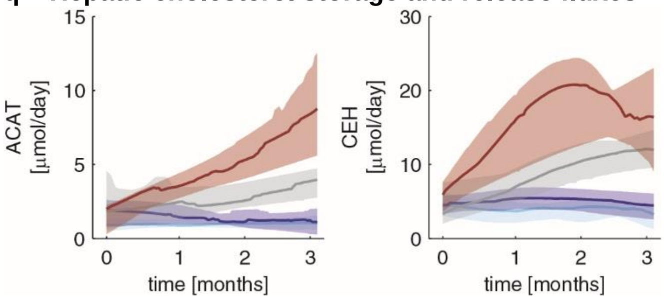

r – Bile acid fluxes

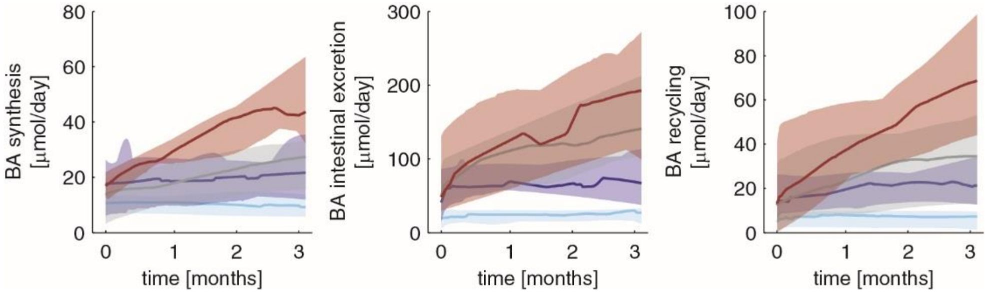

s – Intestinal metabolic fluxes

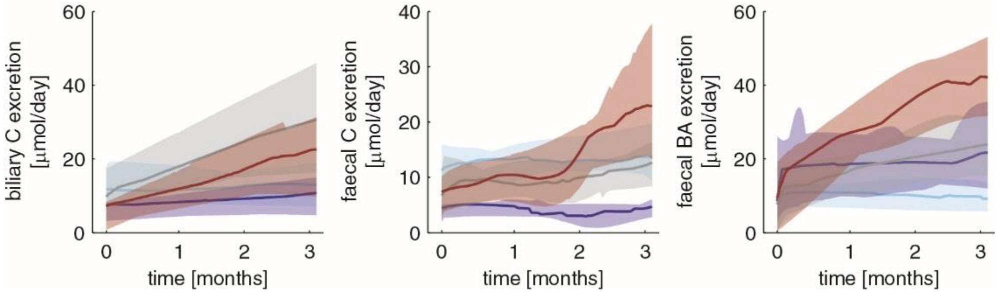

t – Respiratory fluxes

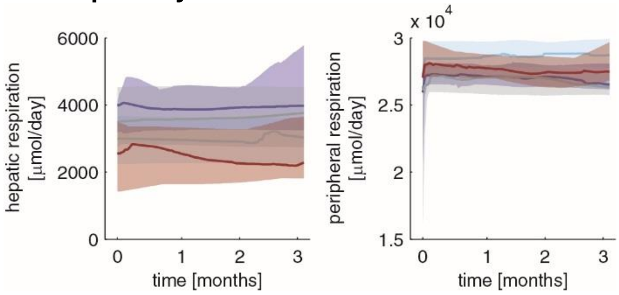

S1 Fig.: Predicted metabolite pools and flux trajectories.
Panels **a-b** display the dynamics in metabolite pools over time and panels **e-t** display the corresponding flux trajectories. We selected the n=100 best trajectories (top 10% based on WSSE). The 10% range around the median trajectory is depicted by the shaded area and the median trajectory for each model component is depicted by the solid line for the low-fat diet group (light blue), high-fat diet group (dark blue), non-dyslipidemic Metabolic Syndrome phenotype (gray) and the dyslipidemic Metabolic Syndrome phenotype (red) respectively. Experimental data is represented by the black error bars (mean ± standard deviation).
