# Supplementary material for: In vivo and in silico dynamics of the development of Metabolic Syndrome
Source: PLoS Comput Biol. 2018 Jun 7;14(6):e1006145. doi: 10.1371/journal.pcbi.1006145 (PMC5991635; doi:10.1371/journal.pcbi.1006145)
Supplement: S1 Note — (PDF) [file pcbi.1006145.s002.pdf]

S1 Note: Experimental setup

Supplemental material for “*In vivo* and *in silico* dynamics of the development of Metabolic Syndrome” by Y.J.W. Rozendaal, Y. Wang, Y. Paalvast, L.L. Tambyrajah, Z. Li, K. Willems van Dijk, P.C.N. Rensen, J.A. Kuivenhoven, A.K. Groen, P.A.J. Hilbers, N.A.W. van Riel

In this study we used male APOE*3-Leiden.CETP mice on a high-fat, cholesterol containing diet to study the onset and development of diet-induced Metabolic Syndrome. All data is available in S1 Data.

Choice of animal model

The APOE*3-Leiden.CETP mouse is a double transgenic animal. Through the genetic APOE*3-Leiden (E3L) mutation, E3L mice have a defective triglyceride-rich lipoprotein (TRL) clearance, which mimics the lower TRL clearance in humans. The E3L mice display an elevated basal cholesterol level and exhibit a human-like lipoprotein profile, develop atherosclerosis upon saturated fat and cholesterol feeding [1,2] and also respond in a human-like manner to drugs used in the treatment of cardiovascular diseases. [3–6]

Another important difference between the murine and human lipoprotein metabolic system is the exchange and transfer of cholesteryl esters (CE) and triglycerides (TG) between HDL and (V)LDL particles through the cholesteryl ester transfer protein (CETP). Mice do not naturally possess the CETP gene and do therefore not respond to HDL-modulating interventions. By cross-breeding the E3L mice to mice expressing the human CETP gene [7], E3L.CETP mice show an even more human-like lipoprotein metabolism. The expression of the human CETP gene shifts the distribution of cholesterol from HDL towards (V)LDL. [1,8] E3L.CETP mice have the ability to reproduce obesity, diabetes, dyslipidemia and atherosclerosis and they respond to both lipid-lowering and HDL-raising interventions. [9–12]

Previous studies using E3L.CETP mice revealed that male E3L.CETP mice upon feeding a high-fat diet develop obesity and diabetes. [10,13] Female E3L.CETP mice that are fed with a high-fat diet with additional cholesterol (the so-called ‘Western-type diet’) develop dyslipidemia and atherosclerosis. [1,9,12,14–21]

The E3L.CETP mouse model can be used to study the associated processes in MetS in more depth. We expected this animal model to develop obesity, diabetes and dyslipidemia upon feeding male E3L.CETP mice a high-fat diet with additional cholesterol.

Study outline

All animal experiments were performed in accordance with the regulations of Dutch law on animal welfare, and the Animal Ethics Committee of the Leiden University Medical Center, Leiden, The Netherlands. Homozygous CETP transgenic mice expressing the human CETP gene under the control of its natural flanking regions (Jackson Laboratory, Bar Harbor, Maine, USA) [7] were crossbred with heterozygous APOE*3-Leiden (E3L) mice [22] to obtain E3L.CETP mice in our own animal facility.

Dietary induction protocol

In this study we used male E3L.CETP transgenic mice that were housed under standard conditions with a 12 h light/dark cycle (7AM-7PM), co-housed with 1-4 animals in conventional cages with free access to food and water, unless indicated otherwise. Animals were housed in a temperature-controlled environment (21 °C). At the age of 10.8 ± 2.2 w weeks, randomized according to body weight and plasma lipids (total cholesterol and triglyceride) and glucose level, mice were divided into three groups: mice were fed a low-fat diet (LFD; n=8), high-fat diet (HFD; n=12) or a high-fat diet with supplemental cholesterol (Sigma, 0.25 gm%) (HFD+C; n=8) for 12 weeks. The low-fat diet has a 20% energy content derived from lard and contains 3.8 kcal/g diet; the high-fat diets have a 60% energy content derived from lard and contains 5.2 kcal/g diet (OpenSource Diets, Research Diets, Inc. New Brunswick, USA). The specific composition of each diet is listed in Table A.

Table A: Composition of the low-fat, high-fat and high-fat with cholesterol diets.

	LFD		HFD		HFD+C	
	gm%	kcal%	gm%	kcal%	gm%	kcal%
protein	19	20	26	20	26	20
carbohydrates	67	70	26	20	26	20
fat	4	10	35	60	35	60
cholesterol	0	0	0.028	0	0.25	0
kcal/g	3.8		5.2		5.2	

Responders and non-responders

Only animals correctly expressing the genotype and responding to the high-fat diet (if applicable) were included in this study. We used the following selection criteria: 1) correct animal genotype (expressing the APOE*3-Leiden and CETP genes) and phenotype: baseline measurement (animals on chow-diet, hence before the start of the dietary induction): plasma triglycerides ≥ 1 mM and plasma total cholesterol ≥ 2 mM; and 2) responding to dietary induction: bodyweight ≥ 35 g after 8 weeks of HFD.

Experimental details of the measurement protocol

During the study, body weight and food intake were measured weekly (Fig. 1a-c). Body composition (lean and fat mass) was determined in conscious mice using an EchoMRI-100 (EchoMRI, Houston, Texas, USA) every other week.
Before (0) and 4, 8, 12 weeks after dietary induction, blood samples were taken by tail vein bleeding into heparin and paraoxon (to inhibit lipase activity) coated capillary tubes, after 5 h of fasting with food withdrawn at 8.00 AM. The tubes were placed on ice and centrifuged, and the obtained plasma was snap frozen in liquid nitrogen and stored at -20°C until further measurements. Plasma was analyzed for cholesterol, triglycerides, lipoproteins, glucose and insulin (Fig. 1d-h).

Plasma metabolites

Blood plasma was assessed for glucose using Glucose reagent 1 and 2 (start reagent) (Instruchemie, Delfzijl, The Netherlands) with a 1 mg/mL glucose standard (Sigma-Aldrich, Saint Louis, Missouri, USA). Insulin was measured using the Ultra Sensitive Mouse Insulin ELISA Kit (Crystal Chem, Downers Grove, Illinois, USA).

Besides the monthly 5 h fasting values, also oral glucose tolerance tests (OGTT) have been performed before (0) and after 6 and 11 weeks dietary induction in overnight fasted mice (7PM-9AM). The glucose (Fig. A a-c) and insulin (Fig. A d-f) responses were measured at 0, 5, 18, 35, 60 and 120 minutes after oral gavage (1 g glucose per kg body weight). Blood glucose is determined by tail bleeding using a portable glucometer (2 µL) and a half of capillary of blood (30 µL) is collected by tail bleeding for insulin measurements.

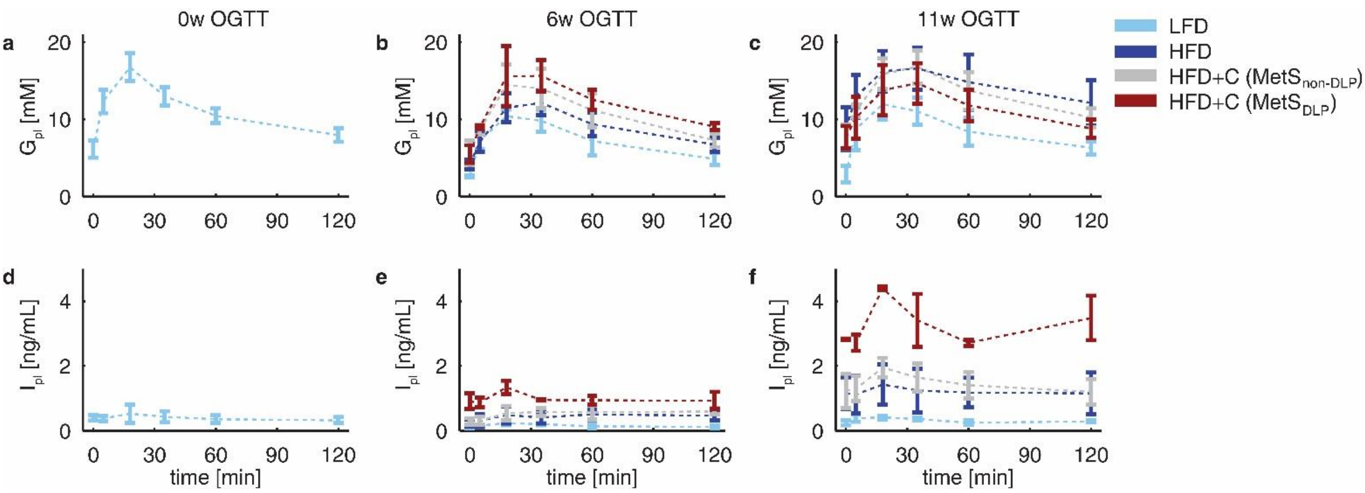

Figure A: Glucose (a-c) and insulin (d-f) response profiles to an Oral Glucose Tolerance Test after 0 (a,d), 6 (b,e) and 11 (c,f) weeks of dietary induction.
The data are depicted using error bars representing mean ± standard deviation for each subgroup separately.

Circulating lipid species were also assessed monthly after 5 hours of fasting. Plasma triglycerides (TG) were measured using a total triglyceride set (Roche diagnostics) using precimat glycerol of 2.29 mM. Plasma free fatty acids were measured using the NEFA-HR(2) kit from Wako Diagnostics (Instruchemie, Delfzijl, The Netherlands). Plasma total cholesterol (TC) was measured using a total cholesterol set (Roche diagnostics) using a cholesterol calibrator of 200 mg/dL (Instruchemie, Delfzijl, The Netherlands). Plasma HDL cholesterol was measured using precipitation of ApoB-containing lipoproteins with PEG 6000#.

Hepatic *de novo* lipogenesis

Hepatic *de novo* lipogenesis (DNL) was assessed at the 3 month time point using a labelled acetate tracer experiment (Fig. 5d). Animals receive drinking water containing sodium-1-^{13}C-acetate (2%) during the final 24 hours of the study (started at 8 AM). To subject the animals to a postprandial fast, food was removed the next morning at 8 AM while the acetate containing drinking water remained available. At 10 AM the

animals were sacrificed by CO_2 inhalation and livers were quickly exercised and stored for further lipid analysis.

Liver histology score

Paraffin-embedded liver sections were stained with hematoxylin and eosin (H&E). According to the histological NAFLD scoring system for rodent models of Liang *et al.* [23] two key features of NASH: steatosis and inflammation, were determined. Briefly, steatosis was scored by hepatocellular vesicular steatosis, i.e. macrovesicular steatosis and microvesicular steatosis fractions separately. Inflammation was scored by analyzing the amount of inflammatory cell aggregates per field using a 100 X magnification (view size of 1.46 mm^2) (Fig. B).

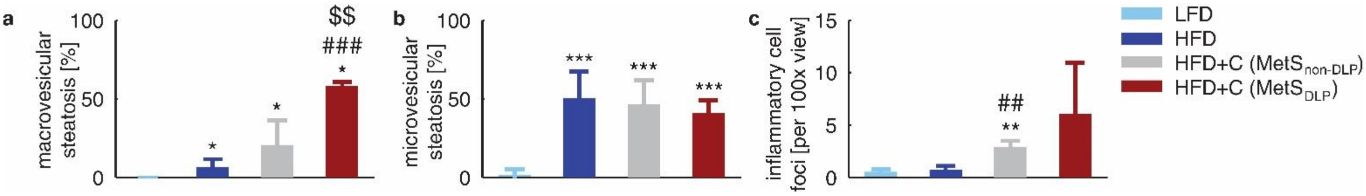

Figure B: Liver histology data of steatosis (a-b) and inflammation markers (c).
The data are depicted using error bars representing mean ± standard deviation for each subgroup separately.
Differences between groups were determined using one-way ANOVA test. When significant differences were found, Fisher's LSD test was used as a post hoc test to determine the differences between two independent groups:
* P<0.05; ** P<0.01; *** P<0.001 as compared to LFD
# P<0.05; ## P<0.01; ### P<0.001 as compared to HFD
$ P<0.05; $$ P<0.01; $$$ P<0.001 as compared to HFD+C ($MetS_{non\text{-}DLP}$).

Liver metabolites

At the end of the 3 months dietary induction experiment, mice were sacrificed and perfused with ice-cold saline via the heart. The livers were isolated and the liver weight was assessed. Liver lipids were extracted according to a modified protocol from Bligh and Dyer [24]. Briefly, small liver pieces were homogenized in ice-cold methanol. After centrifugation, lipids were extracted after addition of 1800 µl $CH_3OH:CHCl_3$ (1:3 v/v) to 45 µl homogenate, followed by vigorous vortexing and phase separation by centrifugation (5 minutes at 2,000 rpm). The $CHCl_3$ phase was dried and dissolved in 2% Triton X-100. TG and TC concentration were measured as described above. Free cholesterol (FC) and cholesteryl ester (CE) concentrations were measured using a commercial kit (Cholesterol/Cholesteryl Ester Quantitation Kit, BioVision, USA). Liver lipids (Fig. 6) were expressed as nmol/mg liver tissue.

Hepatic gene expression analysis

Total RNA was extracted from liver pieces using TriPure isolation reagent (Roche Applied Science, Indianapolis, IN) according to the manufacturer's instructions. RNA quality was examined by the lab-on-a-chip method using Experion Std Sens analysis kit (Biorad, Hercules, CA) and RNA concentration was determined by Nanodrop 1000 spectrophotometer (Thermo-Fischer Scientific). Subsequently, total RNA was reverse-transcribed with RevertAid™ M-MuLV Reverse Transcriptase (Promega, Madison, WI, USA). Quantitative real-time PCR was performed on a CFX96 machine (Bio-Rad, California), the reaction mixture consisting of SYBR-Green Sensimix (QT615, GC Biotech), cDNA, primers (Biolegio, Nijmegen, The Netherlands; see Table B for primer sequences), and nuclease-free water in a total reaction volume of 10 µl. Expression of the selected transcripts was normalized to mRNA levels of hypoxanthine ribosyltransferase (*Hprt*) and cyclophilin (*Cyclo*). Data were calculated as fold difference as compared with the LFD group and are presented in Table C.

Table B: Primer sequences used for RT-qPCR.

Gene	Forward primer	Reverse primer
ACC2	AGATGGCCGATCAGTACGTC	GGGGACCTAGGAAAGCAATC
ACOX1	TATGGGATCAGCCAGAAAGG	ACAGAGCCAAGGGTCACATC
CTP1a	GAGACTTCCAACGCATGACA	ATGGGTTGGGGTGATGTAGA
Cyclo	*CAAATGCTGGACCAAACACAA*	GCCATCCAGCCATTCAGTCT
CYP7A1	CAGGGAGATGCTCTGTGTTCA	AGGCATACATCCCTTCCGTGA
CYP27A1	TCTGGCTACCTGCACTTCCT	CTGGATCTCTGGGCTCTTTG
DGAT2	TCGCGAGTACCTGATGTCTG	CTTCAGGGTGACTGCGTTCT
FASN	GCGCTCCTCGCTTGTCGTCT	TAGAGCCCAGCCTTCCATCTCCTG
FXR	GGCCTCTGGGTACCACTACA	ACATCCCCATCTCTTTGCAC
Hprt	*TTGCTCGAGATGTCATGAAGGA*	*AGCAGGTCAGCAAAGAACTTATAG*
PPARα	ATGCCAGTACTGCCGTTTTC	GGCCTTGACCTTGTTCATGT
SREBP1c	AGCCGTGGTGAGAAGCGCAC	ACACCAGGTCCTTCAGTGATTTGCT

Table C: Relative mRNA expression.

Values are presented as mean value ± standard deviation and have been calculated as fold difference with respect to the mean expression value of the LFD group.

Differences between groups were determined using one-way ANOVA test. When significant differences were found, Fisher's LSD test was used as a post hoc test to determine the differences between two independent groups:

* P<0.05; ** P<0.01; *** P<0.001 as compared to LFD

# P<0.05; ## P<0.01; ### P<0.001 as compared to HFD

$ P<0.05; $$ P<0.01; $$$ P<0.001 as compared to HFD+C ($\text{MetS}_{\text{non-DLP}}$)

Processes/fluxes		LFD	HFD	HFD+C ($\text{MetS}_{\text{non-DLP}}$)	HFD+C (MetS_{DLP})
bile acid synthesis	CYP7A1	1 ± 0.50	4.41 ± 2.07*	6.08 ± 2.76**	8.20 ± 5.25***,#
	CYP27A1	1 ± 0.20	1.19 ± 0.11	1.07 ± 0.24	0.80 ± 0.23#
de novo lipogenesis	FASN	1 ± 0.62	0.49 ± 0.13	0.46 ± 0.36*	0.76 ± 0.36
	ACC2	1 ± 0.42	0.80 ± 0.22	0.52 ± 0.23*	0.81 ± 0.21
	DGAT2	1 ± 0.09	1.21 ± 0.37	0.23 ± 0.05***,###	0.20 ± 0.08***,###
fatty acid β-oxidation	ACOX1	1 ± 0.20	1.24 ± 0.39	1.43 ± 0.48*	1.41 ± 0.39
	CPT1a	1 ± 0.29	2.16 ± 0.67**	2.24 ± 0.94**	2.19 ± 0.69*
nuclear transcript factors	FXR	1 ± 0.21	1.48 ± 0.39*	1.62 ± 0.41**	1.67 ± 0.46*
	SREBP1c	1 ± 0.29	0.99 ± 0.30	1.11 ± 0.48	0.84 ± 0.03
	PPARα	1 ± 0.21	1.95 ± 0.37***	2.05 ± 0.39***	2.30 ± 0.70***

References

1. Westerterp M, van der Hoogt CC, de Haan W, Offerman EH, Dallinga-Thie GM, Jukema JW, et al. Cholesteryl ester transfer protein decreases high-density lipoprotein and severely aggravates atherosclerosis in APOE*3-Leiden mice. Arterioscler Thromb Vasc Biol. 2006;26: 2552–2559. doi:10.1161/01.ATV.0000243925.65265.3c
2. Zadelaar S, Kleemann R, Verschuren L, de Vries-Van der Weij J, van der Hoorn J, Princen HM, et al. Mouse models for atherosclerosis and pharmaceutical modifiers. Arterioscler Thromb Vasc Biol. 2007;27: 1706–1721. doi:10.1161/ATVBAHA.107.142570
3. Delsing DJM, Offerman EH, Duyvenvoorde W van, Boom H van der, Wit ECM de, Gijbels MJJ, et al. Acyl-CoA:Cholesterol Acyltransferase Inhibitor Avasimibe Reduces Atherosclerosis in Addition to Its Cholesterol-Lowering Effect in ApoE*3-Leiden Mice. Circulation. 2001;103: 1778–1786. doi:10.1161/01.CIR.103.13.1778
4. van der Hoorn JWA, Kleemann R, Havekes LM, Kooistra T, Princen HMG, Jukema JW. Olmesartan and pravastatin additively reduce development of atherosclerosis in APOE*3Leiden transgenic mice. J Hypertens. 2007;25: 2454–2462. doi:10.1097/HJH.0b013e3282ef79f7
5. Kleemann R, Princen HMG, Emeis JJ, Jukema JW, Fontijn RD, Horrevoets AJG, et al. Rosuvastatin reduces atherosclerosis development beyond and independent of its plasma cholesterol-lowering effect in APOE*3-Leiden transgenic mice: evidence for antiinflammatory effects of rosuvastatin. Circulation. 2003;108: 1368–1374. doi:10.1161/01.CIR.0000086460.55494.AF
6. Kooistra T, Verschuren L, de Vries-van der Weij J, Koenig W, Toet K, Princen HMG, et al. Fenofibrate reduces atherogenesis in ApoE*3Leiden mice: evidence for multiple antiatherogenic effects besides lowering plasma cholesterol. Arterioscler Thromb Vasc Biol. 2006;26: 2322–2330. doi:10.1161/01.ATV.0000238348.05028.14
7. Jiang XC, Agellon LB, Walsh A, Breslow JL, Tall A. Dietary cholesterol increases transcription of the human cholesteryl ester transfer protein gene in transgenic mice. Dependence on natural flanking sequences. J Clin Invest. 1992;90: 1290–1295. doi:10.1172/JCI115993
8. Rensen PCN, Havekes LM. Cholesteryl ester transfer protein inhibition: effect on reverse cholesterol transport? Arterioscler Thromb Vasc Biol. 2006;26: 681–684. doi:10.1161/01.ATV.0000214979.24518.95
9. de Haan W, de Vries-van der Weij J, van der Hoorn JWA, Gautier T, van der Hoogt CC, Westerterp M, et al. Torcetrapib does not reduce atherosclerosis beyond atorvastatin and induces more proinflammatory lesions than atorvastatin. Circulation. 2008;117: 2515–2522. doi:10.1161/CIRCULATIONAHA.107.761965
10. van den Hoek AM, van der Hoorn JWA, Maas AC, van den Hoogen RM, van Nieuwkoop A, Droog S, et al. APOE*3Leiden.CETP transgenic mice as model for pharmaceutical treatment of the metabolic syndrome. Diabetes Obes Metab. 2014;16: 537–544. doi:10.1111/dom.12252
11. van der Hoogt CC, de Haan W, Westerterp M, Hoekstra M, Dallinga-Thie GM, Romijn JA, et al. Fenofibrate increases HDL-cholesterol by reducing cholesteryl ester transfer protein expression. J Lipid Res. 2007;48: 1763–1771. doi:10.1194/jlr.M700108-JLR200
12. van der Hoorn JWA, de Haan W, Berbée JFP, Havekes LM, Jukema JW, Rensen PCN, et al. Niacin increases HDL by reducing hepatic expression and plasma levels of cholesteryl ester transfer protein in APOE*3Leiden.CETP mice. Arterioscler Thromb Vasc Biol. 2008;28: 2016–2022. doi:10.1161/ATVBAHA.108.171363
13. van Dam AD, Nahon KJ, Kooijman S, van den Berg SM, Kanhai AA, Kikuchi T, et al. Salsalate activates brown adipose tissue in mice. Diabetes. 2015;64: 1544–1554. doi:10.2337/db14-1125
14. Auvinen HE, Wang Y, Princen H, Romijn JA, Havekes LM, Smit JWA, et al. Both transient and continuous corticosterone excess inhibit atherosclerotic plaque formation in APOE*3-leiden.CETP mice. PloS One. 2013;8: e63882. doi:10.1371/journal.pone.0063882
15. Berbée JFP, Boon MR, Khedoe PPSJ, Bartelt A, Schlein C, Worthmann A, et al. Brown fat activation reduces hypercholesterolaemia and protects from atherosclerosis development. Nat Commun. 2015;6. doi:10.1038/ncomms7356
16. van der Hoorn JWA, Jukema JW, Havekes LM, Lundholm E, Camejo G, Rensen PCN, et al. The dual PPARalpha/gamma agonist tesaglitazar blocks progression of pre-existing atherosclerosis in APOE*3Leiden.CETP transgenic mice. Br J Pharmacol. 2009;156: 1067–1075. doi:10.1111/j.1476-5381.2008.00109.x
17. Kühnast S, van der Tuin SJL, van der Hoorn JWA, van Klinken JB, Simic B, Pieterman E, et al. Anacetrapib reduces progression of atherosclerosis, mainly by reducing non-HDL-cholesterol, improves lesion stability and adds to the beneficial effects of atorvastatin. Eur Heart J. 2015;36: 39–48. doi:10.1093/eurheartj/ehu319
18. Li Z, Wang Y, van der Sluis RJ, van der Hoorn JWA, Princen HMG, Van Eck M, et al. Niacin reduces plasma CETP levels by diminishing liver macrophage content in CETP transgenic mice. Biochem Pharmacol. 2012;84: 821–829. doi:10.1016/j.bcp.2012.06.020
19. Wang Y, Berbée JFP, Stroes ES, Smit JWA, Havekes LM, Romijn JA, et al. CETP expression reverses the reconstituted HDL-induced increase in VLDL. J Lipid Res. 2011;52: 1533–1541. doi:10.1194/jlr.M016659
20. Wang Y, van der Tuin SJL, Tjeerdema N, Bieghs V, Rensen SS, Fu J, et al. Plasma cholesteryl ester transfer protein: a biomarker for hepatic macrophages. Novel modulators of lipoprotein metabolism : implications for steatohepatitis and atherosclerosis. 2013.
21. Wang Y, Parlevliet ET, Geerling JJ, van der Tuin SJL, Zhang H, Bieghs V, et al. Exendin-4 decreases liver inflammation and atherosclerosis development simultaneously by reducing macrophage infiltration. Br J Pharmacol. 2014;171: 723–734. doi:10.1111/bph.12490
22. van den Maagdenberg AM, Hofker MH, Krimpenfort PJ, de Bruijn I, van Vlijmen B, van der Boom H, et al. Transgenic mice carrying the apolipoprotein E3-Leiden gene exhibit hyperlipoproteinemia. J Biol Chem. 1993;268: 10540–10545.
23. Liang W, Menke AL, Driessen A, Koek GH, Lindeman JH, Stoop R, et al. Establishment of a general NAFLD scoring system for rodent models and comparison to human liver pathology. PloS One. 2014;9: e115922. doi:10.1371/journal.pone.0115922
24. Bligh EG, Dyer WJ. A rapid method of total lipid extraction and purification. Can J Biochem Physiol. 1959;37: 911–917. doi:10.1139/o59-099
