# Supplementary material for: In vivo and in silico dynamics of the development of Metabolic Syndrome
Source: PLoS Comput Biol. 2018 Jun 7;14(6):e1006145. doi: 10.1371/journal.pcbi.1006145 (PMC5991635; doi:10.1371/journal.pcbi.1006145)
Supplement: S2 Note — (PDF) [file pcbi.1006145.s003.pdf]

S2 Note: Detailed model description of MINGLeD

Supplemental material for “*In vivo* and *in silico* dynamics of the development of Metabolic Syndrome” by Y.J.W. Rozendaal, Y. Wang, Y. Paalvast, L.L. Tambyrajah, Z. Li, K. Willems van Dijk, P.C.N. Rensen, J.A. Kuivenhoven, A.K. Groen, P.A.J. Hilbers, N.A.W. van Riel

MINGLeD (Model Integrating Glucose and Lipid Dynamics) is a dynamic, computational model that describes Metabolic Syndrome phenotypes in terms of clinical symptoms and co-morbidities. It comprises of the pathways that are necessary to describe energy metabolism from dietary intake to storage and exchange between tissues. Metabolite pools have been lumped to obtain a comprehensive model that has sufficient level of detail to describe the interactions and regulations occurring in a healthy metabolic system, but also in different Metabolic Syndrome phenotypes. A scheme of the metabolite pools and fluxes is given in Fig. A. MINGLeD uses a whole body approach and includes metabolically active tissues. The multi-compartmental design comprises of the plasma, liver, intestinal lumen and peripheral tissues. The main contributors to the metabolic pathways in the periphery are considered to be adipose tissue and skeletal

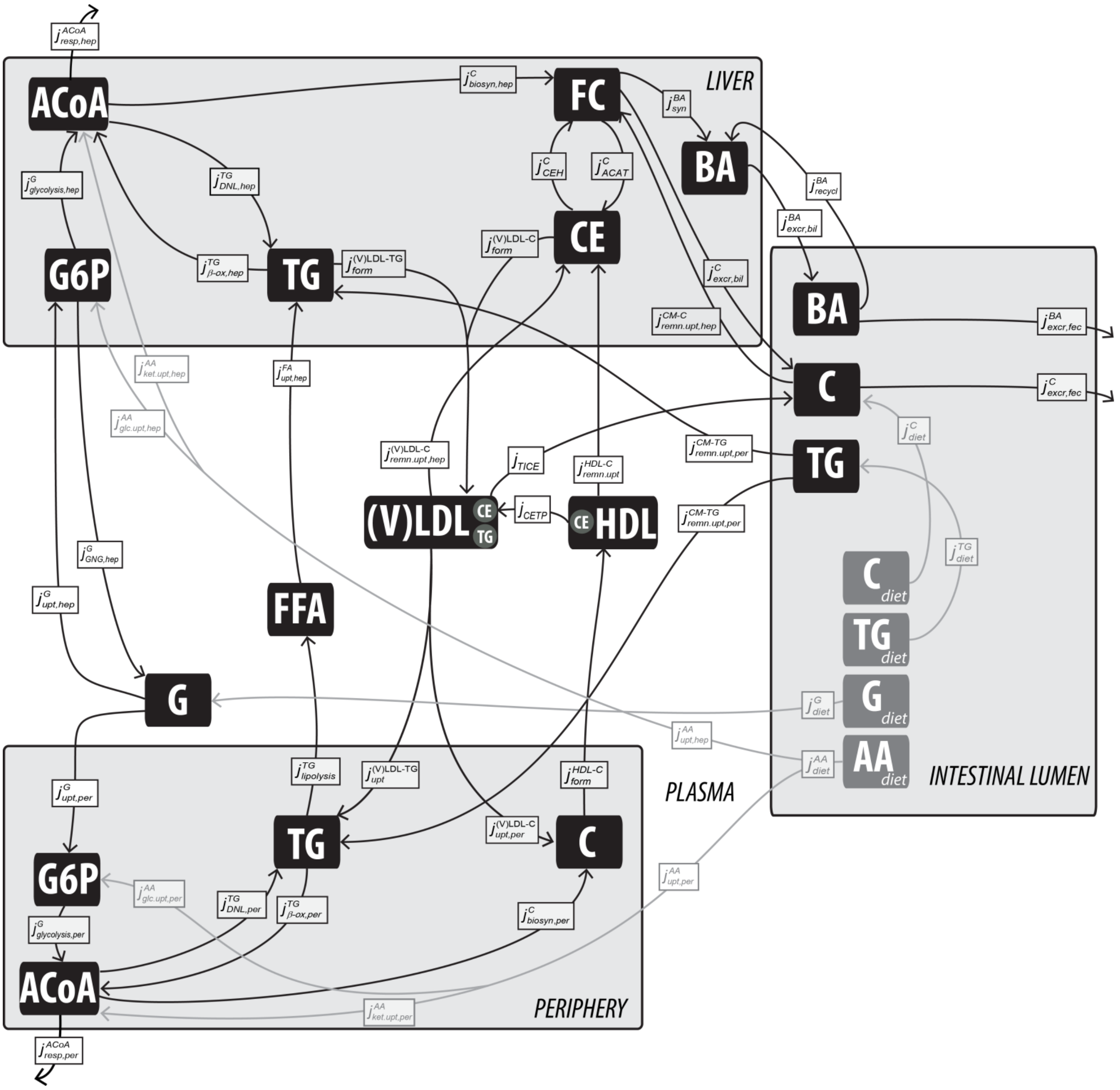

Figure A: Schematic overview of the computational model MINGLeD.
This multi-compartment framework encompasses pathways in dietary absorption, hepatic, peripheral and intestinal lipid metabolism, hepatic and plasma lipoprotein metabolism and plasma, hepatic and peripheral carbohydrate metabolism. The metabolite pools in the different tissue compartments are displayed in the black frames; the corresponding metabolic fluxes are represented using the arrows. The gray fluxes represent the dietary inflow in terms of the different macronutrients derived from the experimental data. See Tables A and B for a description of the metabolic states and fluxes. ACoA, Acetyl CoA; BA, bile acid; C, cholesterol; CE, cholesteryl ester; FC, free cholesterol; FFA, free fatty acid; G6P, glucose-6-phosphate; HDL, high density lipoprotein; TG, triglyceride; (V)LDL, (very) low density lipoprotein.

muscle. MINGLeD includes pathways for carbohydrate, lipid and cholesterol metabolism, and computes pool sizes (concentrations) of various metabolites present in these systems. Table A lists which metabolites are included in MINGLeD in each of the metabolically active tissues. Acetyl coenzyme A (ACoA) is the central intermediate metabolite interconnecting the carbohydrate and lipid metabolic pathways. The metabolic fluxes are based on first order mass action kinetics and listed in Table B. The type of equations is similar to those used in previous ADAPT studies [1–4]. These lumped flux and reaction equations were derived using previous (biological) knowledge about the processes were are modelling. Since the underlying regulation is often unknown (especially for lipid species), we refrained from composing our flux equations using stoichiometry coefficients. Therefore the underlying regulation is not explicatively modelled, but will be inferred by the time-dependent parameters using ADAPT.

MINGLeD aims to describe the metabolic pathways of the day's average. Therefore specific (detailed) pathways for neither the postprandial phase, nor the fasting state have been included. The overall metabolic fluxes are able to describe the day's average in a consistent manner, and describe both healthy metabolic states and various stages of metabolic derailment of the system as is present in the Metabolic Syndrome. Note that insulin has not been included since insulin synthesis, secretion and action are on a much shorter time scale (minutes) than we use the model for (day's average).

Below we provide a detailed description of the modelled metabolites, how they are biologically regulated, the motivation to include these in the computational model, and how these interactions and pathways have been implemented in a system of Ordinary Differential Equations (ODEs) to describe mass balance during steady state.

Macronutrient intake

The dietary intake is specified in the form of carbohydrates (in MINGLeD referred to as glucose substrates), fat (in MINGLeD referred to as triglycerides substrates), cholesterol and proteins. All these macronutrients are included such that the energy intake of the complete diet is taken into account. The values corresponding the dietary intake for the different diets are listed Table A in S1 Note.

In the model glucose is taken up into the plasma from the dietary glucose directly. Fat and cholesterol are packed into chylomicrons (CM). These undergo triglyceride hydrolysis and are delivered to the hepatic and peripheral tissues; the chylomicron remnants that contain cholesterol are delivered to the liver. Note that chylomicrons are not explicitly included in MINGLeD since we do not aim to create a specific model describing the postprandial phase. Including the chylomicron particles would not improve the ability of the model to describe the available experimental data.

Although the metabolic pathways of amino acid metabolism have not been included in MINGLeD, we do take dietary protein intake into account since 20% of the energy of the diet is derived from protein sources. The amino acids (AA) are taken up by the liver and the periphery, and in each of these tissues they can undergo either glucogenic or ketogenic metabolism.

Glucose metabolism

Plasma glucose metabolism is controlled by glucose inflow from the diet, consecutive glucose uptake by liver and periphery and gluconeogenesis (GNG) in the liver. Insulin-independent, glucose-concentration independent glucose uptake by the brain and erythrocytes was not taken into account since these tissues were not explicitly specified, and no experimental data of these fluxes is available. Instead the brain and erythrocytes are part of the periphery since this compartment comprises of all other tissues apart from liver, intestine and plasma.

Glucose is trapped in the form of glucose-6-phosphate (G6P), and can be retrieved in the plasma through GNG in the liver. Glycogen pools were omitted since glycogenolysis and glycogenesis would both be connected to G6P, and on a day's average basis would not be separable (only the net effect is modelled).

The amino acids (AA) of which the dietary protein is composed of are converted to G6P via the glucogenic pathway and to acetyl coenzyme A (ACoA) via the ketogenic pathway in both liver and periphery. We impose that both pathways contribute equally to metabolism of the dietary protein uptake.

It is worth mentioning that all these pathways have been lumped and therefore no intermediates in e.g. the glycolysis pathway have been included. This yields a compact model of which the majority of the modelled

variables can be estimated from the data with accuracy. If we would include detailed pathways with many intermediates, which cannot be coupled to experimental data, an identifiable model cannot be obtained, i.e. these intermediate metabolites and fluxes do not provide accurate predictions and may present unphysiological behavior.

$$\frac{dG_{pl}}{dt} = j^{G}_{diet} + j^{G}_{GNG,hep} - j^{G}_{upt,hep} - j^{G}_{upt,per} \qquad \left(\frac{dx_1}{dt}\right)$$

$$\frac{dG6P_{hep}}{dt} = j^{G}_{upt,hep} + j^{AA}_{glc.upt,hep} - j^{G}_{glycolysis,hep} - j^{G}_{GNG,hep} \qquad \left(\frac{dx_9}{dt}\right)$$

$$\frac{dG6P_{per}}{dt} = j^{G}_{upt,per} + j^{AA}_{glc.upt,per} - j^{G}_{glycolysis,per} \qquad \left(\frac{dx_{14}}{dt}\right)$$

Plasma lipid metabolism

Free fatty acids (FFA) – also called non-esterified fatty acids (NEFA) – are carboxylic acids with long hydrocarbon chains. These FFA differ by length and saturation, ranging from short chain (≤5 carbons) up to very long chain fatty acids (≥22 carbons). We do not consider these different chain lengths as different species; we have lumped the fatty acid to be one pool that will inherently comprise all these different types of fatty acids.

Circulating free fatty acids originate after lipolysis of peripheral triglycerides through lipoprotein lipase (LPL) activity. Fatty acids are taken up from the plasma by the liver, where they are stored in the form of triglycerides (TG).

$$\frac{dFFA_{pl}}{dt} = 3 \cdot j^{TG}_{lipolysis} - j^{FA}_{upt,hep} \qquad \left(\frac{dx_2}{dt}\right)$$

Plasma lipoprotein metabolism

Lipoproteins are particles that have a hydrophobic core with a surrounding hydrophilic layer. This makes them ideal for the transport of triglycerides and cholesterol through the circulation. They are traditionally classified based on density, size, apolipoprotein composition and origin of synthesis. The density of a lipoprotein is determined by the amount of protein and lipid the particle contains. A higher density indicates that the lipoprotein has a higher ratio of protein to lipid content. HDL is the smallest and most dense particle.

Commonly, a distinction into five classes is made: chylomicrons (CM), very low density lipoproteins (VLDL), intermediate density lipoproteins (IDL), low density lipoproteins (LDL) and high density lipoproteins (HDL). In MINGLeD we distinguish between high density lipoproteins that carry cholesterol (HDL-C) and (very) low density lipoproteins that carry both cholesterol ((V)LDL-C) and triglycerides ((V)LDL-TG). All endogenously derived triglyceride-rich lipoproteins (TRL) have been packed under the name of (V)LDL, but inherently comprise of very low density (VLDL), intermediate density (IDL) and low density (LDL) lipoproteins.

Note that HDL-TG has not been included since it is generally known that the majority of the molecules within HDL can be contributed to cholesterol particles and the TG content is negligible. Furthermore, the ratio between TG and cholesterol in HDL is not known (and not experimentally assessed).

HDL-C is formed from pre-HDL which originates from the periphery and is packed with cholesterol that has been esterified through lecithin-cholesterol acyltransferase (LCAT). Plasma HDL-C is subjected to plasma lipid transfer upon action of the cholesteryl ester transfer protein (CETP), and the remaining remnant particles are taken up by the liver via scavenger receptor class B1 (SR-B1). CETP collects triglycerides from TRL in exchange for cholesteryl esters from HDL and vice versa. However, since we did not include HDL-TG, MINGLeD only considers the cholesterol transfer from HDL-C to (V)LDL-C. Note that the rate equation for CETP was chosen to be dependent on plasma TG pools, since this is generally considered to be the driver behind CETP action.

HDL-C also plays an important role in the reverse cholesterol transport (RCT) pathway: the transport of cholesterol from the peripheral tissues back to the liver, after which cholesterol can be secreted via the bile into the feces. The pathways in MINGLeD allow for this reverse cholesterol transport to take place.

(V)LDL is assembled in the liver from the hepatic triglyceride and cholesteryl ester pool, and then secreted into the plasma. Circulating (V)LDL is being lipolyzed and thereby delivers triglycerides and cholesterol to peripheral tissues. The remaining remnant particles are TG depleted and are taken up by LDL receptor-mediated uptake by the liver, where they are recycled into the cholesteryl ester pool.

(V)LDL can also undergo transport to the intestinal lumen through transintestinal cholesterol excretion (TICE). The TICE rate equation was chosen to be dependent on the VLDL-C pool. The plasma compartments contributing to TICE are not completely known, and may be both coming from ApoB-containing lipoproteins as well as from erythrocytes, it was decided to make it dependent on VLDL-C only, since erythrocytes are not included in the model.

$$\frac{dHDLC_{pl}}{dt} = j_{form}^{HDLC} - j_{CETP} - j_{remn.upt}^{HDLC} \qquad \left(\frac{dx_3}{dt}\right)$$

$$\frac{d(V)LDLC_{pl}}{dt} = j_{form}^{(V)LDLC} + j_{CETP} - j_{remn.upt,hep}^{(V)LDLC} - j_{upt,per}^{(V)LDLC} - j_{TICE} \qquad \left(\frac{dx_4}{dt}\right)$$

$$\frac{d(V)LDLTG_{pl}}{dt} = j_{form}^{(V)LDLTG} - j_{upt}^{(V)LDLTG} \qquad \left(\frac{dx_5}{dt}\right)$$

Hepatic lipid metabolism

The hepatic triglyceride pool is supplied by chylomicron remnant uptake, fatty acid uptake from the plasma, *de novo* lipogenesis (DNL; synthesis of triglycerides from ACoA substrates). Details about the reaction stoichiometry can be found in Table B. The triglyceride pool is drained by β-oxidation and for the assembly of (V)LDL particles.

Acetyl coenzyme A is a species that participates in many different metabolic processes. It plays a role in carbohydrate, lipid and cholesterol metabolism. It originates from breakdown of carbohydrate substrates (glycolysis) and from breakdown of fatty acids (β-oxidation).
It is used as a substrate for *de novo* lipogenesis of triglycerides and for the biosynthesis of cholesterol (through the mevalonate pathway). It can also be oxidized via the citric acid cycle, yielding ATP.

$$\frac{dTG_{hep}}{dt} = j_{remn.upt,hep}^{CMTG} + \frac{j_{upt,hep}^{FA}}{3} + j\,\frac{j_{DNL,hep}^{TG}}{21.4} - j_{\beta ox,hep}^{TG} - j_{form}^{(V)LDLTG} \qquad \left(\frac{dx_6}{dt}\right)$$

$$\frac{dACoA_{hep}}{dt} = j_{ket.upt,hep}^{AA} + 2\cdot j_{glycolysis,hep}^{G} + 21.4\cdot j_{\beta ox,hep}^{TG} - j_{DNL,hep}^{TG} - j_{biosyn,hep}^{C} - j_{resp,hep}^{ACoA} \qquad \left(\frac{dx_{10}}{dt}\right)$$

Hepatic cholesterol metabolism

We distinguish between free cholesterol and cholesteryl ester as pools of cholesterol present in the liver (both species have been measured separately in the hepatic cholesterol pool).
The hepatic free cholesterol pool is supplied by chylomicron remnant uptake, cholesterol biosynthesis and by cholesteryl ester hydrolase (CEH; hydrolysis of CE to FC) activity. The FC pool is drained by Acyl-coenzyme A:cholesterol acyltransferase (ACAT; esterification of FC to CE) activity and biliary cholesterol excretion. Hepatic free cholesterol is also a precursor for bile acid synthesis.

The hepatic cholesteryl ester pool is supplied by (V)LDL-C and HDL-C remnant uptake and esterification of free cholesterol through ACAT. Cholesteryl esters are drained from the liver for (V)LDL-C assembly and by hydrolysis through CEH.

$$\frac{dFC_{hep}}{dt} = j_{remn.upt}^{CMC} + \frac{j_{biosyn,hep}^{C}}{13.5} + j_{CEH}^{C} - j_{ACAT}^{C} - j_{syn}^{BA} - j_{excr,bil}^{C} \qquad \left(\frac{dx_7}{dt}\right)$$

$$\frac{dCE_{hep}}{dt} = j_{remn.upt,hep}^{(V)LDLC} + j_{remn.upt,hep}^{HDLC} + j_{ACAT}^{C} - j_{form}^{(V)LDLC} - j_{CEH}^{C} \qquad \left(\frac{dx_8}{dt}\right)$$

Hepatic bile acid metabolism

Bile acids are synthesized in the liver from endogenous cholesterol and can be secreted into the lumen of the intestine. Because of their amphipathic properties they are able to emulsify dietary lipids and they thereby facilitate lipid absorption. The majority of the intestinal bile acids will be recycled by the enterohepatic circulation. The bile acids can be taken up into the circulation, return to the liver and be re-secreted.

$$\frac{dBA_{hep}}{dt} = j_{synt,hep}^{BA} + j_{recycl}^{BA} - j_{excr,bil}^{BA} \qquad \left(\frac{dx_{11}}{dt}\right)$$

Peripheral lipid metabolism
Many pathways in peripheral lipid metabolism resemble those of hepatic lipid metabolism. Triglycerides are included in the peripheral compartment by chylomicron remnant uptake, TG uptake resulting from lipolyzed (V)LDL and *de novo* lipogenesis. When triglycerides undergo lipolysis – by activity of lipoprotein lipase (LPL) – they are released in the plasma in the form of free fatty acids. Triglycerides are also removed from the peripheral TG pool by β-oxidation.

The peripheral ACoA pool is determined by many processes that yield ACoA particles: the ketogenic uptake of dietary proteins, from carbohydrate substrates originating after glycolysis and from lipid substrates originating after the β-oxidation of triglycerides.
ACoA is a substrate for both lipid production (DNL) and for cholesterol production (biosynthesis). It can also be oxidized to generate energy.

$$\frac{dTG_{per}}{dt} = j^{CMTG}_{remn.upt,per} + j^{(V)LDLTG}_{upt} + \frac{j^{TG}_{DNL,per}}{21.4} - j^{TG}_{lipolysis} - j^{TG}_{\beta ox,per} \qquad \left(\frac{dx_{12}}{dt}\right)$$

$$\frac{dACoA_{per}}{dt} = j^{AA}_{ket.upt,per} + 2 \cdot j^{G}_{glycolysis,per} + 21.4 \cdot j^{TG}_{\beta ox,per} - j^{TG}_{DNL,per} - j^{C}_{biosyn,per} - j^{ACoA}_{resp,per} \qquad \left(\frac{dx_{15}}{dt}\right)$$

Peripheral cholesterol metabolism
In the peripheral compartment, MINGLeD does not discriminate between free cholesterol and cholesteryl ester particles as no experimental data on the cholesterol pools in these tissues is available. Therefore we consider the peripheral cholesterol pool to be referred to as the total cholesterol content present in the periphery.
The cholesterol present in the HDL-C particles is retrieved from the peripheral cholesterol pool. The cholesterol pool is replenished by (V)LDL-C uptake and cholesterol biosynthesis from ACoA. ACoA is the common precursor that links TG and cholesterol synthesis and exists not only in the liver, but in every tissue (adipose tissue also expresses HMG-CoA reductase, which is the limiting step in cholesterol biosynthesis).

$$\frac{dC_{per}}{dt} = j^{(V)LDLC}_{upt,per} + \frac{j^{C}_{biosyn,per}}{13.5} - j^{HDLC}_{form} \qquad \left(\frac{dx_{13}}{dt}\right)$$

Intestinal metabolism
In the intestinal lumen, MINGLeD distinguishes between triglycerides, cholesterol and bile acids. Triglycerides and cholesterol originating from the diet are packed into chylomicrons that deliver these components to the liver and peripheral tissues. Biliary excretion from the liver delivers cholesterol and bile acids to the luminal compartment. Bile acids can be re-absorbed into the liver. Additional cholesterol originating from (V)LDL enters the intestine through transintestinal cholesterol excretion (TICE). Both bile acids and cholesterol can be eliminated from the system through fecal excretion.

$$\frac{dTG_{int}}{dt} = j^{TG}_{diet} - j^{CMTG}_{remn.upt,hep} - j^{CMTG}_{remn.upt,per} \qquad \left(\frac{dx_{16}}{dt}\right)$$

$$\frac{dC_{int}}{dt} = j^{C}_{diet} + j^{C}_{excr,bil} + j_{TICE} - j^{CMC}_{remn.upt} - j^{C}_{excr,fec} \qquad \left(\frac{dx_{17}}{dt}\right)$$

$$\frac{dBA_{int}}{dt} = j^{BA}_{excr,bil} - j^{BA}_{excr,fec} - j^{BA}_{recycl} \qquad \left(\frac{dx_{18}}{dt}\right)$$

Table A: Metabolite pools as modelled states described in MINGLeD.
All metabolite pools in MINGLeD are expressed in µmol.

G_{pl}	plasma glucose	(x_1)
FFA_{pl}	plasma free fatty acids	(x_2)
$HDLC_{pl}$	plasma HDL-C	(x_3)
$(V)LDLC_{pl}$	plasma (V)LDL-C	(x_4)
$(V)LDLTG_{pl}$	plasma (V)LDL-TG	(x_5)
TG_{hep}	hepatic triglycerides	(x_6)
FC_{hep}	hepatic free cholesterol	(x_7)
CE_{hep}	hepatic cholesterol ester	(x_8)
$G6P_{hep}$	hepatic glucose-6-phosphate	(x_9)
$ACoA_{hep}$	hepatic Acetyl CoA	(x_{10})
BA_{hep}	hepatic bile acids	(x_{11})
TG_{per}	peripheral triglycerides	(x_{12})
C_{per}	peripheral cholesterol	(x_{13})
$G6P_{per}$	peripheral glucose-6-phosphate	(x_{14})
$ACoA_{per}$	peripheral Acetyl CoA	(x_{15})
TG_{int}	intestinal triglycerides	(x_{16})
C_{int}	intestinal cholesterol	(x_{17})
BA_{int}	intestinal bile acids	(x_{18})

Table B: List of included flux pathways in MINGLeD.
All fluxes in MINGLeD are expressed in µmol/day.

Dietary fluxes

j_{diet}^{G}	$G_{diet} \rightarrow G_{pl}$	dietary glucose intake flux *	(j_1)
j_{diet}^{TG}	$TG_{diet} \rightarrow TG_{int}$	dietary triglyceride intake flux *	(j_2)
j_{diet}^{C}	$C_{diet} \rightarrow C_{int}$	dietary cholesterol intake flux *	(j_3)
j_{diet}^{AA}	$AA_{diet} \rightarrow AA_{hep} + AA_{per}$	dietary protein intake flux *	(j_4)

Macronutrient uptake fluxes

$j_{remn.upt,hep}^{CMTG} = k_{remn.upt,hep}^{CMTG} \cdot TG_{int}$	$TG_{\mathrm{int}} \rightarrow TG_{hep}$	hepatic chylomicron remnant uptake	(j_5)
$j_{remn.upt,per}^{CMTG} = k_{remn.upt,per}^{CMTG} \cdot TG_{int}$	$TG_{\mathrm{int}} \rightarrow TG_{per}$	peripheral chylomicron remnant uptake	(j_6)
$j_{remn.upt,hep}^{CMC} = k_{remn.upt,hep}^{CMC} \cdot C_{int}$	$C_{\mathrm{int}} \rightarrow FC_{hep}$	hepatic chylomicron remnant uptake	(j_7)
$j_{upt,hep}^{AA} = k_{upt,hep}^{AA} \cdot j_{diet}^{AA}$	$AA_{diet} \rightarrow AA_{hep}$	hepatic protein uptake	(j_8)
$j_{upt,per}^{AA} = k_{upt,per}^{AA} \cdot j_{diet}^{AA}$	$AA_{diet} \rightarrow AA_{per}$	peripheral protein uptake	(j_9)
$j_{glc.upt,hep}^{AA} = 0.5 \cdot j_{upt,hep}^{AA}$ #	$AA_{hep} \rightarrow G6P_{hep}$	hepatic glucogenic protein uptake	(j_{10})
$j_{glc.upt,per}^{AA} = 0.5 \cdot j_{upt,per}^{AA}$ #	$AA_{per} \rightarrow G6P_{per}$	peripheral glucogenic protein uptake	(j_{11})
$j_{ket.upt,hep}^{AA} = 0.5 \cdot j_{upt,hep}^{AA}$ #	$AA_{hep} \rightarrow ACoA_{hep}$	hepatic ketogenic protein uptake	(j_{12})
$j_{ket.upt,hep}^{AA} = 0.5 \cdot j_{upt,per}^{AA}$ #	$AA_{per} \rightarrow ACoA_{per}$	peripheral ketogenic protein uptake	(j_{13})
$j_{upt,hep}^{G} = k_{upt,hep}^{G} \cdot G_{pl}$	$G_{pl} \rightarrow G6P_{hep}$	hepatic glucose uptake	(j_{14})
$j_{upt,per}^{G} = k_{upt,per}^{G} \cdot G_{pl}$	$G_{pl} \rightarrow G6P_{per}$	peripheral glucose uptake	(j_{15})

Carbohydrate fluxes

$j_{glycolysis,hep}^{G} = k_{glycolysis,hep}^{G} \cdot G6P_{hep}$	$1 \cdot G6P_{hep} \rightarrow 2 \cdot ACoA_{hep}$ (a)	hepatic glycolysis	(j_{16})
$j_{GNG.hep}^{G} = k_{GNG,hep}^{G} \cdot G6P_{hep}$	$G6P_{hep} \rightarrow G_{pl}$	gluconeogenesis	(j_{17})
$j_{glycolysis,per}^{G} = k_{glycolysis,per}^{G} \cdot G6P_{per}$	$1 \cdot G6P_{hep} \rightarrow 2 \cdot ACoA_{per}$ (a)	peripheral glycolysis	(j_{18})

Lipoprotein fluxes

$j_{upt}^{(V)LDLTG} = k_{upt}^{(V)LDLTG} \cdot (V)LDLTG_{pl}$	$(V)LDLTG_{pl} \rightarrow TG_{per}$	peripheral (V)LDL-TG uptake	(j_{19})
$j_{upt,per}^{(V)LDLC} = k_{upt,per}^{(V)LDLC} \cdot (V)LDLC_{pl}$	$(V)LDL\_C_{pl} \rightarrow C_{per}$	peripheral (V)LDL-C uptake	(j_{20})
$j_{remn.upt,hep}^{(V)LDLC} = k_{remn.upt,hep}^{(V)LDLC} \cdot (V)LDLC_{pl}$	$(V)LDLC_{pl} \rightarrow CE_{hep}$	hepatic (V)LDL-C remnant uptake	(j_{21})
$j_{form}^{(V)LDLTG} = k_{form}^{(V)LDLTG} \cdot TG_{hep}$	$TG_{hep} \rightarrow (V)LDLTG_{pl}$	recruitment of triglycerides for (V)LDL assembly	(j_{22})
$j_{form}^{(V)LDLC} = k_{form}^{(V)LDLC} \cdot CE_{hep}$	$CE_{hep} \rightarrow (V)LDLC_{pl}$	recruitment of cholesterol particles for (V)LDL assembly	(j_{23})

$j_{form}^{HDLC} = k_{form}^{HDLC} \cdot C_{per}$	$C_{per} \rightarrow HDLC_{pl}$	HDL-C formation	(j_{24})
$j_{remn.upt}^{HDLC} = k_{remn.upt}^{HDLC} \cdot HDLC_{pl}$	$HDLC_{pl} \rightarrow CE_{hep}$	hepatic HDL-C remnant uptake	(j_{25})
$j_{TICE} = k_{TICE} \cdot (V)LDLTG_{pl}$	$(V)LDLC_{pl} \rightarrow C_{int}$	transintestinal cholesterol excretion	(j_{26})
$j_{CETP} = k_{CETP} \cdot (V)LDLTG_{pl}$	$HDLC_{pl} \rightarrow (V)LDLC_{pl}$	cholesterol ester transfer protein flux: exchange of cholesterol derived from HDL to (V)LDL	(j_{27})

Lipid fluxes

$j_{upt,hep}^{FA} = k_{upt,hep}^{FA} \cdot FFA_{pl}$	$3 \cdot FFA_{pl} \rightarrow 1 \cdot TG_{hep}$ (b)	hepatic fatty acid uptake	(j_{28})
$j_{\beta ox,hep}^{TG} = k_{\beta ox,hep} \cdot TG_{hep}$	$1 \cdot TG_{hep} \rightarrow 21.4 \cdot ACoA_{hep}$ (c)	hepatic beta-oxidation	(j_{29})
$j_{biosyn,hep}^{C} = k_{biosyn,hep} \cdot ACoA_{hep}$	$13.5 \cdot ACoA_{hep} \rightarrow 1 \cdot FC_{hep}$ (d)	hepatic cholesterol biosynthesis	(j_{30})
$j_{DNL,hep}^{TG} = k_{DNL,hep} \cdot ACoA_{hep}$	$21.4 \cdot ACoA_{hep} \rightarrow 1 \cdot TG_{hep}$ (c)	hepatic de novo lipogenesis	(j_{31})
$j_{lipolysis}^{TG} = k_{LPL} \cdot TG_{per}$	$1 \cdot TG_{per} \rightarrow 3 \cdot FFA_{pl}$ (b)	peripheral lipolysis	(j_{32})
$j_{\beta ox,per}^{TG} = k_{\beta ox,per} \cdot TG_{per}$	$1 \cdot TG_{per} \rightarrow 21{,}4 \cdot ACoA_{per}$ (c)	peripheral beta-oxidation	(j_{33})
$j_{biosyn,per}^{C} = k_{biosyn,per} \cdot ACoA_{per}$	$13.5 \cdot ACoA_{per} \rightarrow 1 \cdot C_{per}$ (d)	peripheral cholesterol biosynthesis	(j_{34})
$j_{DNL,per}^{TG} = k_{DNL,per} \cdot ACoA_{per}$	$21.4 \cdot ACoA_{per} \rightarrow 1 \cdot TG_{per}$ (c)	peripheral de novo lipogenesis	(j_{35})

Cholesterol fluxes

$j_{ACAT}^{C} = k_{ACAT} \cdot FC_{hep}$	$FC_{hep} \rightarrow CE_{hep}$	hepatic cholesterol storage	(j_{36})
$j_{CEH}^{C} = k_{CEH} \cdot CE_{hep}$	$CE_{hep} \rightarrow FC_{hep}$	hepatic cholesterol release	(j_{37})
$j_{syn}^{BA} = k_{syn}^{BA} \cdot FC_{hep}$	$FC_{hep} \rightarrow BA_{hep}$	bile acid synthesis	(j_{38})
$j_{excr,bil}^{BA} = k_{excr,bil}^{BA} \cdot BA_{hep}$	$BA_{hep} \rightarrow BA_{\mathrm{int}}$	biliary bile acid excretion	(j_{39})
$j_{recycl}^{BA} = k_{recycl}^{BA} \cdot BA_{int}$	$BA_{\mathrm{int}} \rightarrow BA_{hep}$	bile acid recycling	(j_{40})
$j_{excr,bil}^{C} = k_{excr,bil}^{C} \cdot FC_{hep}$	$FC_{hep} \rightarrow C_{int}$	biliary cholesterol excretion	(j_{41})

Removal fluxes

$j_{excr,fec}^{C} = k_{excr,fec}^{C} \cdot C_{int}$	$C_{int} \rightarrow \varnothing$	fecal cholesterol excretion	(j_{41})
$j_{excr,fec}^{BA} = k_{excr,fec}^{BA} \cdot BA_{int}$	$BA_{int} \rightarrow \varnothing$	fecal bile acid excretion	(j_{42})
$j_{resp,hep}^{ACoA} = k_{resp,hep} \cdot ACoA_{hep}$	$ACoA_{hep} \rightarrow \varnothing$	hepatic Acetyl CoA respiration	(j_{43})
$j_{resp,per}^{ACoA} = k_{resp,per} \cdot ACoA_{per}$	$ACoA_{hep} \rightarrow \varnothing$	peripheral Acetyl CoA respiration	(j_{44})

* The dietary fluxes are considered as inputs from the experimental data set.
[#] The factor 0.5 originates from our assumption that both the glucogenic and ketogenic pathway contribute equally to metabolism of the dietary protein uptake.

The flux equations are derived using stoichiometry rules:
(a) The breakdown of one molecule of glucose results in two molecules of Acetyl CoA: $2 \cdot G6P \leftrightarrow 1 \cdot ACoA$
(b) Triglycerides are esters derived from glycerol and three fatty acids per particle: $3 \cdot FFA \leftrightarrow 1 \cdot TG$
(c) The composition of triglycerides depends on the length and saturation of the fatty acid chains of which it is composed. The conversion of TG to Acetyl CoA is therefore derived based on energy content. We adjust the number of Acetyl CoA molecules derived from TG assuming that the energy density of body fat equals the energy density of the consumed food. This results in: $1 \cdot TG \leftrightarrow 21.4 \cdot ACoA$
(d) Acetyl CoA ($CH_3COSCoa$) is a two-carbon compound; cholesterol ($C_{27}H_{46}O$) has 27 carbon units: $1 \cdot (F)C \leftrightarrow 13.5 \cdot ACoA$

Implementation details

The mathematical model and optimization procedures were implemented in MATLAB (2013b, The Mathworks, Natick, Massachusetts). The ordinary differential equations were solved with compiled MEX files using numerical integrators from the SUNDIALS CVode package (2.6.0, Lawrence Livermore National Laboratory, Livermore, California) [5]. An absolute and relative tolerance of 10^{-6} was used. The MATLAB nonlinear least-squares solver *lsqnonlin* (from the Optimization Toolbox), which uses an inferior reflective Newton method, was used to estimate model parameters [6]. The termination tolerances for the objective function and the parameter estimates were set to 10^{-8}, the maximum number of iterations allowed was set to 10^{3} and the maximum number of function evaluations allowed to 10^{5}.

References

1. Tiemann CA, Vanlier J, Oosterveer MH, Groen AK, Hilbers PAJ, van Riel NAW. Parameter trajectory analysis to identify treatment effects of pharmacological interventions. PLoS Comput Biol. 2013;9: e1003166. doi:10.1371/journal.pcbi.1003166
2. Tiemann CA, Vanlier J, Hilbers PAJ, van Riel NAW. Parameter adaptations during phenotype transitions in progressive diseases. BMC Syst Biol. 2011;5: 174. doi:10.1186/1752-0509-5-174
3. van Riel NAW, Tiemann CA, Vanlier J, Hilbers PAJ. Applications of analysis of dynamic adaptations in parameter trajectories. Interface Focus. 2013;3: 20120084. doi:10.1098/rsfs.2012.0084
4. Hijmans BS, Tiemann CA, Grefhorst A, Boesjes M, van Dijk TH, Tietge UJF, et al. A systems biology approach reveals the physiological origin of hepatic steatosis induced by liver X receptor activation. FASEB J Off Publ Fed Am Soc Exp Biol. 2015;29: 1153–1164. doi:10.1096/fj.14-254656
5. Hindmarsh AC, Brown PN, Grant KE, Lee SL, Serban R, Shumaker DE, et al. SUNDIALS: Suite of Nonlinear and Differential/Algebraic Equation Solvers. ACM Trans Math Softw. 2005;31: 363–396. doi:10.1145/1089014.1089020
6. Coleman T, Li Y. An Interior Trust Region Approach for Nonlinear Minimization Subject to Bounds. SIAM J Optim. 1996;6: 418–445. doi:10.1137/0806023
