# Supplementary material for: In vivo and in silico dynamics of the development of Metabolic Syndrome
Source: PLoS Comput Biol. 2018 Jun 7;14(6):e1006145. doi: 10.1371/journal.pcbi.1006145 (PMC5991635; doi:10.1371/journal.pcbi.1006145)
Supplement: S3 Note — (PDF) [file pcbi.1006145.s004.pdf]

S3 Note: Data used for model calibration

Supplemental material for "*In vivo* and *in silico* dynamics of the development of Metabolic Syndrome" by Y.J.W. Rozendaal, Y. Wang, Y. Paalvast, L.L. Tambyrajah, Z. Li, K. Willems van Dijk, P.C.N. Rensen, J.A. Kuivenhoven, A.K. Groen, P.A.J. Hilbers, N.A.W. van Riel

Parameters for MINGLeD are estimated to calibrate the model by fitting to experimentally observed data. Maximum likelihood estimation is used to minimize the difference between model output and data. This error is referred to as the cost function and consists of the weighted sum of squared errors (WSSE) between model outputs and data. Table A describes what which model outputs are constraint to which data.

When fitting MINGLeD to separate phenotype snapshots, the cost function is as follows:

$$\chi_j^2 = \sum_{i=1}^{N_i} \left(\frac{y_i(\theta, t_j) - \mu_{i,j}}{\sigma_{i,j}} \right)^2 \qquad \textbf{Eq. 1}$$

with χ_j^2 representing the WSSE at time point j. It evaluates model outputs y_i (listed in Table 1) as predicted by the parameter set θ in comparison to the mean ($\mu_{i,j}$) and standard deviation ($\sigma_{i,j}$) of the corresponding experimentally observed data of metabolite i at time j.

When MINGLeD is fitted over the complete timespan using the ADAPT methodology, the cost function is as follows:

$$\chi_{ADAPT}^2 = \chi^2 + \chi_{reg}^2$$
$$= \sum_{i=1}^{N_i} \sum_{j=1}^{N_t} \left(\frac{y_i(\theta, t_j) - \mu_{i,j}}{\sigma_{i,j}} \right)^2 + \lambda \cdot \sum_{k=1}^{N_p} \left(\frac{\theta_{j,k} - \theta_{j,k-1}}{\theta_{0,k}} \right)^2 \qquad \textbf{Eq. 2}$$

The WSSE (χ^2) was extended and is now evaluated over the complete timespan. Note that the cost function was also extended with a regularization term, weighted by the regularization coefficient λ (which was set to 0.1 in this study) in which N_p is the number of parameters and $\theta_{j,k}$ the parameter k at time j and $\theta_{0,k}$ the initial value of parameter k.

Table A: List of SSE components

observable	respective model output		respective data component	unit
y_1	x_1	G_{pl}	plasma glucose [mM] · estimated plasma volume [mL] [(a)]	μmol
y_2	x_2	FFA_{pl}	plasma FFA data [mM] · estimated plasma volume [mL] [(a)]	μmol
y_3	x_3	$HDLC_{pl}$	plasma HDL-C data [mM] · estimated plasma volume [mL] [(a)]	μmol
y_4	x_5	$(V)LDLTG_{pl}$	plasma TG data [mM] · estimated plasma volume [mL] [(a)]	μmol
y_5	x_4	$(V)LDLC_{pl}$	(plasma total cholesterol data [mM] - plasma HDL-C data [mM]) · estimated plasma volume [mL] [(a)]	μmol
y_6	x_6	TG_{hep}	hepatic TG data [μmol]	μmol
y_7	x_7	FC_{hep}	hepatic FC data [μmol]	μmol
y_8	x_8	CE_{hep}	hepatic TC data [μmol] - hepatic FC data [μmol]	μmol
y_9	x_{31}	$j_{DNL,hep}^{TG}$	$DNL_{C16:0}$ data + $DNL_{C18:0}$ data + $DNL_{C18:1}$ data + $^1/_9 \cdot CE_{C18:0}$ data + $^1/_9 \cdot CE_{C18:1}$ data [μmol/g liver] · liver weight data [g] [(b)]	μmol/day
y_{10}	x_{12}	TG_{per}	(fat mass data [mg] / molar mass of TG[(c)]) $\cdot 10^6$	μmol
y_{11}	$j_8 + j_9$	$j_{upt,hep}^{AA} + j_{upt,per}^{AA}$	dietary protein intake data[(d)]	μmol/day

[(a)] the plasma volume is approximated by V_{pl} = 0.7704 + 0.0117 · BW [1]
[(b)] chain elongation is accounted for as $^1/_9$th of a newly synthesized fatty acid
[(c)] the molar mass of an average triglyceride molecule was assumed to be 853 g/mol [2]
[(d)] the daily dietary protein intake was calculated in terms of glucose equivalent particles, taking into account the 20% of the energy content of the diet is derived from protein, and glucose having an energy density of 4.18 kcal/g and a molar mass of 180 g/mol [2]

References

1. Yen TT, Stienmetz J, Simpson PJ. Blood volume of obese (ob-ob) and diabetic (db-db) mice. Proc Soc Exp Biol Med Soc Exp Biol Med N Y N. 1970;133: 307–308.
2. Desilva PE. Système International (SI) units for plasma, serum, or blood concentrations. Diabetes Care. 2007;30: 769.
